# Distribution and diversity of type VI secretion system clusters in *Enterobacter bugandensis* and *Enterobacter cloacae*

**DOI:** 10.1099/mgen.0.001148

**Published:** 2023-12-06

**Authors:** Amy J. G. Anderson, Becca Morrell, Guillermo Lopez Campos, Miguel A. Valvano

**Affiliations:** ^1^​ Wellcome-Wolfson Institute for Experimental Medicine, Queen’s University Belfast, Belfast, BT9 7BL, UK

**Keywords:** comparative genomics, T6SS effectors, T6SS gene cluster, T6SS gene organization, T6SS subtype

## Abstract

Gram-negative bacteria use type VI secretion systems (T6SSs) to antagonize neighbouring cells. Although primarily involved in bacterial competition, the T6SS is also implicated in pathogenesis, biofilm formation and ion scavenging. *Enterobacter* species belong to the ESKAPE pathogens, and while their antibiotic resistance has been well studied, less is known about their pathogenesis. Here, we investigated the distribution and diversity of T6SS components in isolates of two clinically relevant *Enterobacter* species, *E. cloacae* and *E. bugandensis *. T6SS clusters are grouped into four types (T6SS^i^-T6SS^iv^), of which type i can be further divided into six subtypes (i1, i2, i3, i4a, i4b, i5). Analysis of a curated dataset of 31 strains demonstrated that most of them encode T6SS clusters belonging to the T6SS^i^ type. All T6SS-positive strains possessed a conserved i3 cluster, and many harboured one or two additional i2 clusters. These clusters were less conserved, and some strains displayed evidence of deletion. We focused on a pathogenic *E. bugandensis* clinical isolate for comprehensive *in silico* effector prediction, with comparative analyses across the 31 isolates. Several new effector candidates were identified, including an evolved VgrG with a metallopeptidase domain and a Tse6-like protein. Additional effectors included an anti-eukaryotic catalase (KatN), M23 peptidase, PAAR and VgrG proteins. Our findings highlight the diversity of *Enterobacter* T6SSs and reveal new putative effectors that may be important for the interaction of these species with neighbouring cells and their environment.

## Data Summary

The complete genome sequences of *E. bugandensis* isolates E104107 and E105227 are available under NCBI accession numbers NZ_CP110983.1 and NZ_CP110985.1, respectively. The code used is described in Table S1 (available in the online version of this article), while strain accession numbers and metadata are shown in Table S6. All authors confirm support of data, codes and protocols noted within the article.

**Impact Statement**

The genus *Enterobacter* includes multiple species of Gram-negative bacteria from the family *Enterobacteriaceae *, which are commonly found in the human gut microbiota and can become opportunistic pathogens resulting in bacteraemia, intra-abdominal infections and infections in multiple body sites. A major concern with *Enterobacter* infections is the frequent appearance of multidrug-resistant isolates, for which these bacteria are included in the ESKAPE list of global-threat pathogens requiring last-resort antibiotics. Many Gram-negative bacteria utilize type VI secretion systems (T6SSs) for bacterial-competition and virulence. Understanding how the *Enterobacter* T6SS can be used to manipulate bacteria and eukaryotic cells may provide insights into the pathogenesis of this genus.

## Introduction

Bacteria often live in complex polymicrobial communities where they compete for nutrients and space [1–3]. Many bacteria have evolved strategies to outcompete their neighbours and dominate their environmental niche [4–6]. One such strategy involves the type VI secretion system (T6SS), a bacterial nanomachine that deploys a range of antibacterial effectors [7–9]. The current model of T6SS assembly comprises 13 core components that are required for secretion. The membrane complex (TssJLM) anchors the T6SS to the bacterial membrane and allows docking of a baseplate complex (TssEGFK) encompassing a VgrG-PAAR spike [10, 11]. The Hcp tube is assembled onto a VgrG trimer and is surrounded by a contractile TssBC sheath in extended conformation [12, 13]. Assembly is coordinated by TssA and the sheath contracts to propel the Hcp-VgrG-PAAR puncturing device across the membrane of adjacent target cells [14–16]. Finally, the sheath is disassembled by TssH, an AAA+ ATPase, in preparation for re-firing.

The puncturing device is decorated with a payload of toxic effectors that perform several functions. Effectors are broadly grouped into two categories: evolved effectors and cargo effectors. Evolved effectors are covalently fused, typically as C-terminal extensions, to any of the VgrG, Hcp or PAAR structural components, while cargo effectors are non-covalently attached to any one of the VgrG, Hcp or PAAR components and may also require chaperones for loading or stability [17–20]. Immunity proteins protect attacking cells from self-intoxication by binding and neutralizing own effectors before loading onto the T6SS [21, 22]. These proteins are often encoded by genes adjacent to their corresponding effector genes. T6SS genes encoding structural components, effectors, immunity proteins, regulators and other accessory proteins are normally arranged in clusters; bacteria may have multiple T6SS clusters within their genomes [23–27]. However, not all effectors are encoded within clusters and their genes may be scattered across the genome. Bacteria can display significant diversity in their T6SS genomic organization, with components distributed in clusters, in lone genes or with T6SS-associated genes, such as *hcp*, *PAAR* and *vgrG*, to form auxiliary modules [28–30]. Although primarily utilized for bacterial competition, the T6SS is a versatile system that can also function in pathogenesis, biofilm formation, anti-fungal activity, self-recognition and ion scavenging [31–35].

The T6SS is widely distributed among Gram-negative bacteria, including many pathogenic species [27, 36–39]. Members of the ESKAPE pathogens utilize the T6SS for a range of activities including inter-bacterial competition and host cell invasion [27, 38, 40–44]. *Enterobacter* species are represented in the last ‘E’ of the ESKAPE pathogens, but the taxonomy of *Enterobacter* species is complicated [45, 46]. Currently, there are 56 species within the genus *Enterobacter *, 22 of which are published with accepted names [List of Prokaryotic Names with Standing in Nomenclature (accessed: 7 June 2023); https://lpsn.dsmz.de/genus/Enterobacter] [47]. Although not all species are pathogenic, some have emerged as important nosocomial pathogens and are generally referred to as the *Enterobacter cloacae* complex [48–51]. The *Enterobacter* T6SS has been investigated in *E. cloacae* type strain ATCC 13047, which possesses two T6SS clusters (termed T6SS-1 and T6SS-2) utilized for gut colonization, bacterial competition, biofilm formation and epithelial cell adherence [40]. Only four effectors involved in bacterial competition have been characterized to date in *E. cloacae *; these include Tae4, an anti-bacterial peptidoglycan-targeting effector with amidase activity [41], two anti-bacterial rearrangement hotspot (Rhs) proteins, RhsA and RhsB [52], and a Tle phospholipase [52].

This study aimed to further elucidate the distribution and diversity of T6SSs within *Enterobacter bugandensis* in comparison with *E. cloacae *. Both species were chosen due to their relevance in clinical settings. *E. bugandensis* was first identified and characterized in 2016 from a nosocomial sepsis outbreak [53]. It is highly multi-drug resistant and believed to be the most virulent species within the genus *Enterobacter* [54, 55]. *E. cloacae* is currently the most frequently isolated *Enterobacter* species in healthcare settings [56, 57]. Here, we characterized the T6SS clusters in a curated set of 31 *E. bugandensis* and *E. cloacae* strains. We also focused on the pathogenic clinical isolate, *E. bugandensis* E104107, to extensively characterize, *in silico*, effectors and other components within T6SS clusters. Comparative analysis of the discussed effectors was performed across all strains in this study.

## Methods

### Analytical workflow

**Fig. 1.**
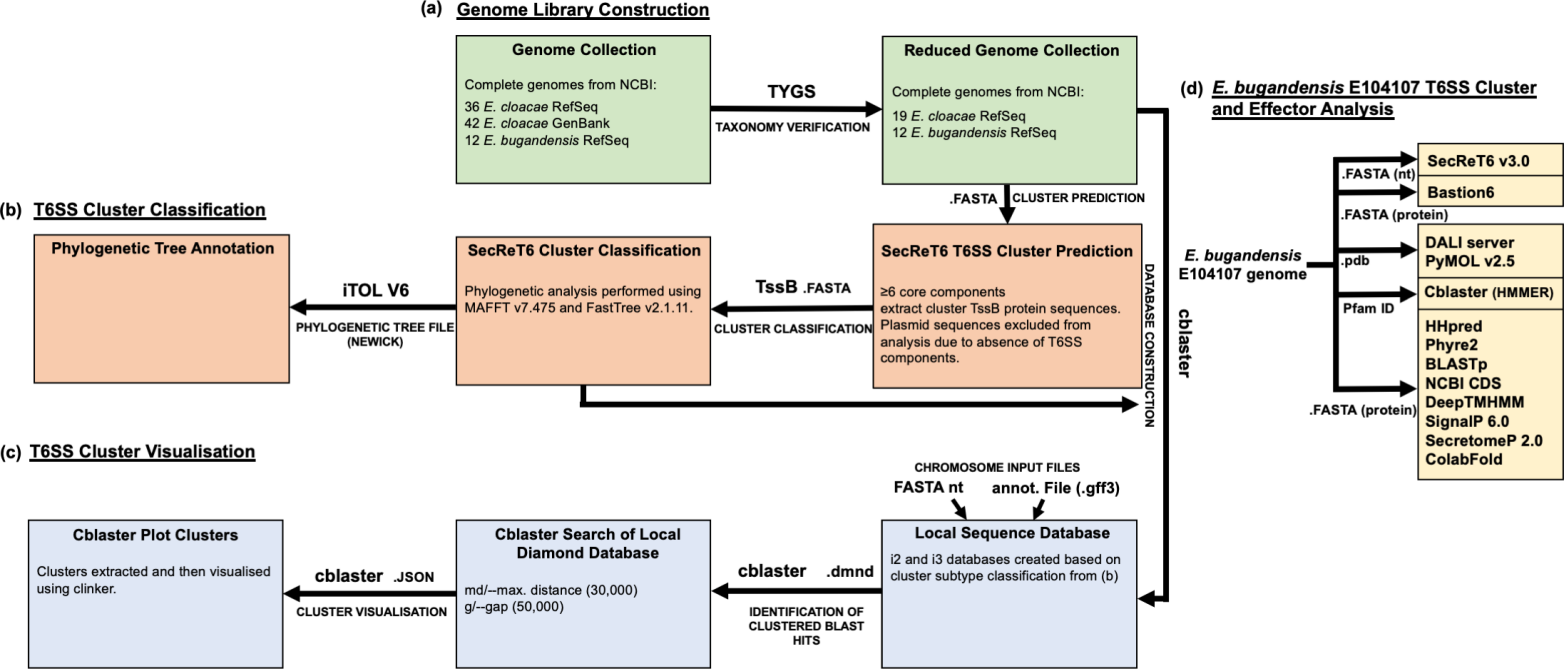
Workflow overview of T6SS analysis in *E. bugandensis* and *E. cloacae* strains. Thirty-one *E. bugandensis* and *E. cloacae* genomes were obtained from NCBI, and the correct taxonomy was confirmed using the Type (Strain) genome server. T6SS clusters were predicted based on their presence of six or more core components. TssB protein sequences from clusters were extracted and submitted to the SecReT6 classification tool for subtype classification. Cblaster was used to plot and visualize clusters across the 31 strains based on cluster subtype. An *E. bugandensis* clinical isolate, *E. bugandensis* E104107, was chosen as a model strain for in-depth T6SS cluster analysis and effector prediction using a range of automated and manual prediction tools. Comparative analysis of identified effector candidates was carried out across the 31 strains included in this study.

The workflow analysis we employed for T6SS cluster extraction, classification, visualization and effector prediction is depicted in Fig. 1.

### Construction of genome libraries

The nucleotide and protein FASTA sequences, and gff3 annotation files for all complete *E. cloacae* and *E. bugandensis* genomes were obtained from the RefSeq NCBI database (18 November 2022) and from GenBank for those not available in the RefSeq database. The correct taxonomy of all 90 extracted genomes was assigned using the Type (Strain) Genome Server (TYGS) [58]. The DNA FASTA query files for all 90 genomes were submitted to TYGS and taxonomy identification was performed using default settings. Incorrectly assigned genomes were removed, leaving us with a curated dataset of 31 *E. bugandensis* and *E. cloacae* genomes.

### Identification, classification and visualization of T6SS clusters

The SecReT6 v3.0 web server was used to identify T6SS components using the chromosome and plasmid FASTA nucleotide files of our curated dataset [59]. The minimum number of T6SS cluster components was set to 6 [29, 60], and all other search parameters were set to default. Plasmids were excluded from further analysis. Extracted TssB protein sequences were compiled and submitted to the SecReT6 v3.0 T6SS classification web server; phylogenetic analysis was performed using MAFFT v7.475 and FastTree v2.1.11 [59]. Cluster subtypes were noted, and the phylogenetic tree file was extracted and submitted to iTOL v6 for annotation and visualization [61].

Cblaster was used to plot and visualize clusters [62]. Local sqlite3, diamond and FASTA genome databases were constructed by cblaster based on cluster subtypes using the FASTA nucleotide and annotation (.gff3) files of the 31 genomes. A local search was performed using the diamond databases and FASTA files containing three conserved protein sequences as queries (Table S2), which included one upstream and downstream flanking gene and a conserved sequence within the cluster. Some clusters demonstrated a conserved upstream flanking region and variable downstream regions. For the clusters with variable downstream regions, one upstream flanking gene and two conserved sequences within the cluster were used. The maximum distance between the start/end of a cluster and an intermediate gene was set to 30 000 bp, the maximum distance between any two hits in a cluster was set to 50 000 bp and intermediate genes were included. The FASTA output file and JSON session file were obtained, and the JSON session file was used to extract the cluster sequences in GenBank (gbk) format. The JSON session file was also used to plot clusters and visualize them in clinker [63], which is also part of cblaster. Clusters were scored and arranged by cblaster based on similarity [62]. Cluster similarity was calculated by *S=h+i.s*, where *h* is the number of query sequences with blast hits, *s* is the number of contiguous gene pairs with conserved synteny and *i* is a weighting factor (default value 0.5) determining the weight of synteny in the similarity score [62].

### *In silico* prediction of T6SS components and effectors

The *E. bugandensis* E104107 genome sequence was used as a template for in-depth *in silico* T6SS analysis and effector prediction. Automated prediction of T6SS components and effectors was performed with SecReT6 v3.0 and Bastion6 web tools, respectively [59, 64]. Genes within and surrounding predicted T6SS clusters, and predicted effectors across the whole genome, were manually analysed by structural (HHpred, Phyre2, DALI server) and sequence similarity [blast, NCBI conserved domain search (CDS)] tools [65–69]. Transmembrane regions and signal peptides were predicted with DeepTMHMM (https://dtu.biolib.com/DeepTMHMM) and SignalP 6.0, respectively [70]. SecretomeP 2.0 was used to predict non-classically secreted proteins [71]. To identify auxiliary modules, a cblaster local FASTA database was created and used to perform HMMER searches using Hcp (PF05638), PAAR (PF05488, PF13665) and VgrG (PF13296, PF04717) Pfam IDs (obtained on 1 March 2023; available at: https://www.ebi.ac.uk/interpro/entry/pfam/#table). PAAR, VgrG and Hcp protein sequences were extracted and NCBI CDS was used to verify correct domain identification. For comparative effector analysis, BLASTp was employed with default parameters to search a local protein FASTA database of the 31 genomes for effectors discussed in this study [68]. ColabFold (combining AlphaFold2 and MMseqs2) was used to predict effector structures from their amino acid sequences, which were visualized with PyMOL v2.5 [72].

## Results

### Most *E. bugandensis and E. cloacae* genomes carry at least two T6SS clusters

**Fig. 2.**
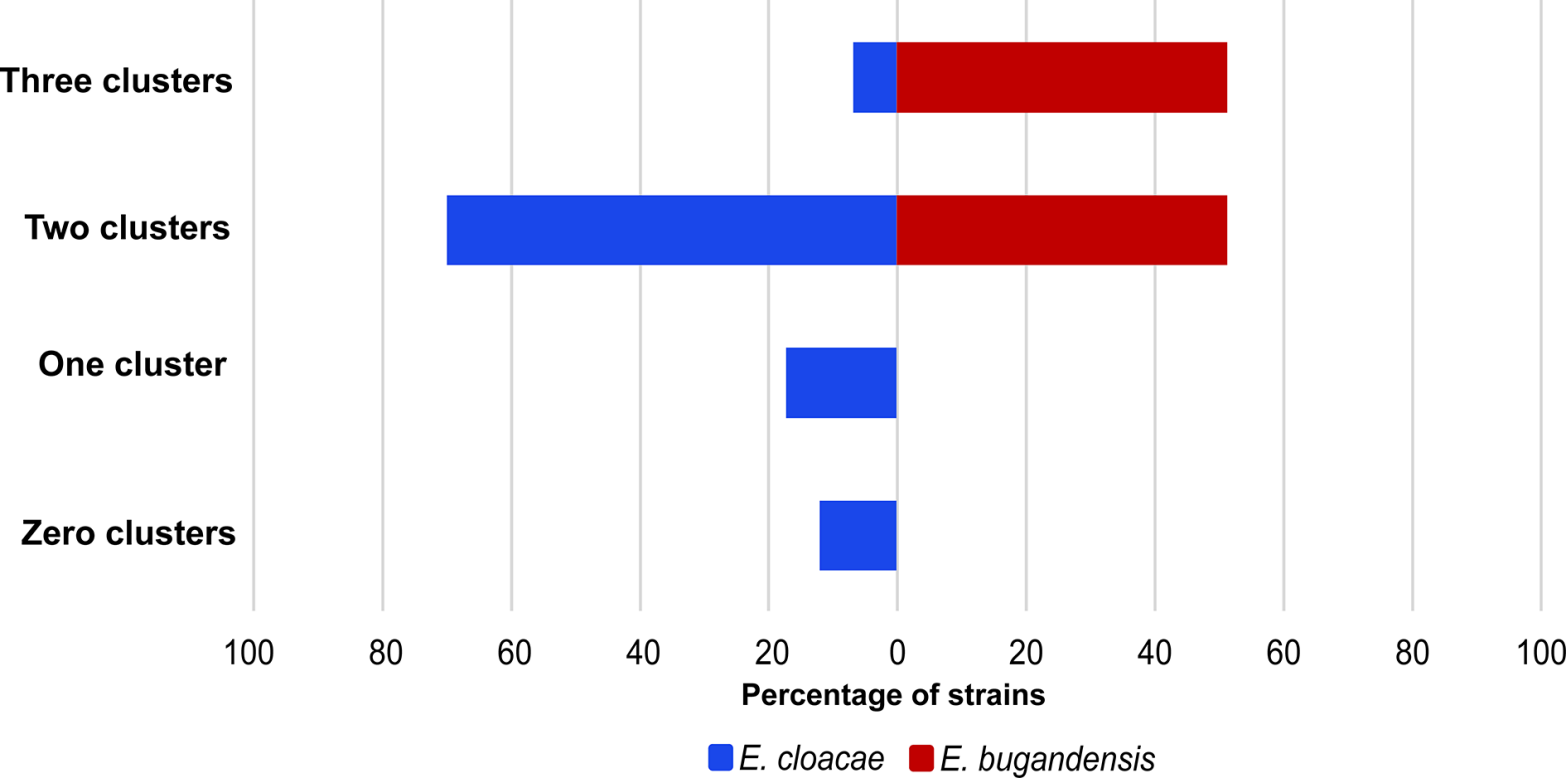
Number of T6SS clusters across *E. bugandensis* and *E. cloacae* strains. The percentage of *E. cloacae* strains possessing 0–3 T6SS clusters is shown in blue. The percentage of *E. bugandensis* strains possessing 0–3 T6SS clusters is shown in red.

We investigated the prevalence and distribution of T6SS clusters in complete genomes of *E. bugandensis* and *E. cloacae* strains using the workflow described in Fig. 1. SecReT6 v3.0 was used to predict effectors encoded in all 31 genomes within our dataset (Table S7). Twelve *E. bugandensis* and 78 *E. cloacae* complete genome sequences were extracted from the NCBI assembly database. Prior to any analysis, we determined *in silico* the taxonomy of all 90 strains using TYGS. From these results, we excluded 59 genomes from our initial dataset since they corresponded to species incorrectly named ‘* E. cloacae *’; these included *Enterobacter sichuanensis *, *E. hormaechei *, *E. asburiae *, *E. mori *, *E. kobei* and *E. roggenkampii *. The remaining 31 genomes, ranging from 4 199 688 to 6 193 009 bp in size (with 3851–6208 proteins, and GC content ranging from 53.73% to 57.26 %), were used for further analysis (Fig. S1). The 31 genomes in our final dataset included 12 *E. bugandensis* and 19 *E. cloacae* strains. T6SS clusters in the 31-genome dataset were identified based on the presence of six or more core gene components, since clusters with fewer than six core components are unlikely to contain the *tssB* gene required for cluster classification (Table S3) [29, 60]. Most *E. bugandensis* and *E. cloacae* strains (93.5%) possessed at least one T6SS cluster (Fig. 2 and Table S4). *E. bugandensis* strains consistently carried two or three T6SS clusters, while *E. cloacae* strains displayed greater variability, ranging from zero (two strains) to three clusters. The plasmids of these strains were excluded from further analysis since they lacked T6SS components, in agreement with a previous study that identified T6SS clusters on only 1 % of 30 660 plasmids [29].

### All T6SS-positive strains carry a primary i3 cluster and a variable number of secondary i2 clusters

**Fig. 3.**
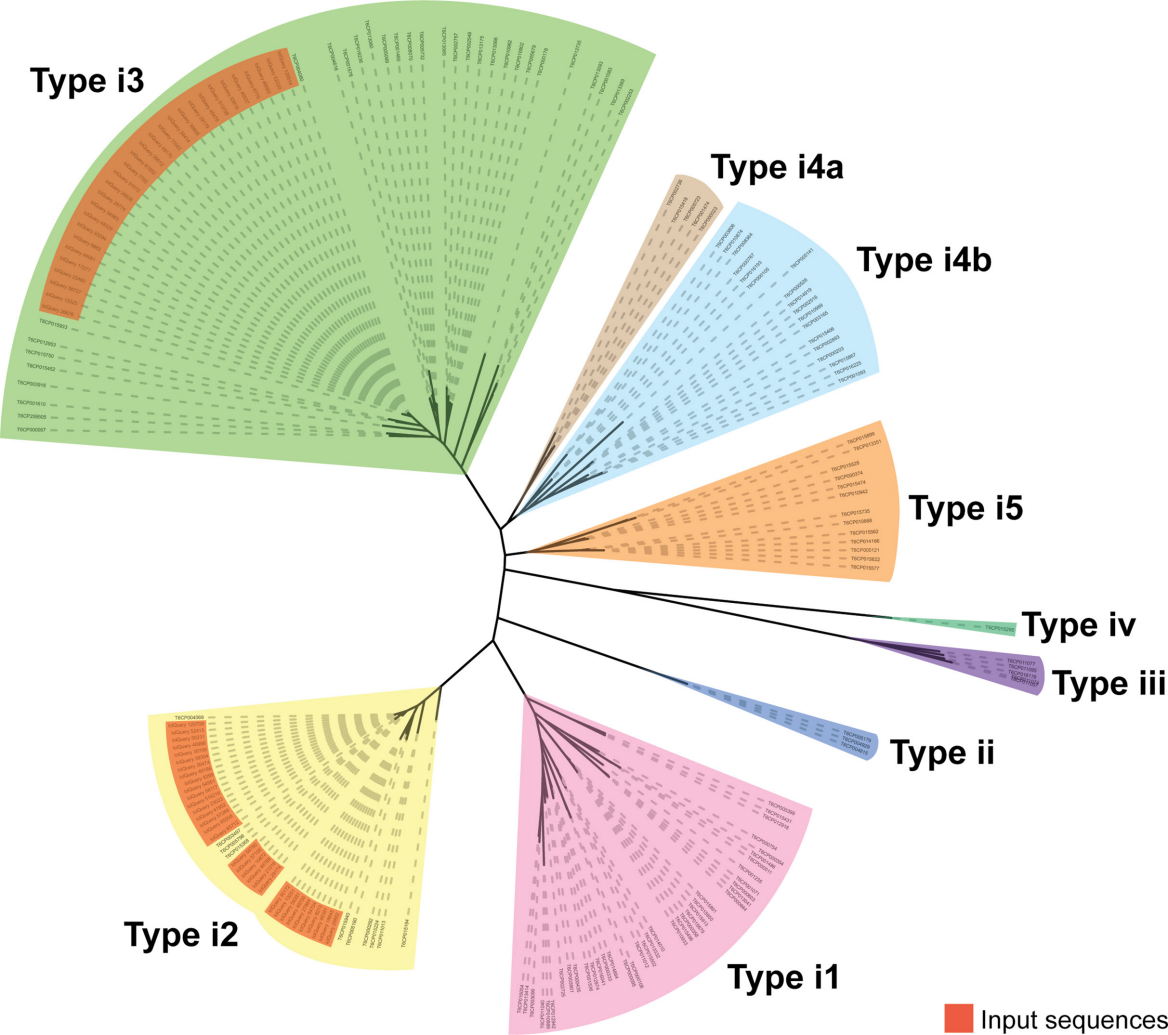
Phylogenetic tree of *E. cloacae* and *E. bugandensis* T6SS cluster classification. A phylogenetic tree of all *E. cloacae* and *E. bugandensis* T6SS clusters identified in this study was reconstructed based on validated TssB protein sequences in the SecReT6 database. Phylogenetic analysis was performed using MAFFT v7.475 and FastTree v2.1.11 via the SecReT6 classification tool. Clusters are divided into four types (i, ii, iii, iv), and type i is further divided into six subtypes (i1, i2, i3, i4a, i4b, i5). All *E. cloacae* and *E. bugandensis* T6SS^i3^ clusters (highlighted in red) are grouped with validated type i3 TssB reference sequences. Similarly, all *E. cloacae* and *E. bugandensis* T6SS^i2^ clusters (highlighted in red) are grouped with validated type i2 TssB reference sequences.

T6SSs can be classified into four types (i, ii, iii, iv), of which T6SS^i^ is the most common type found in the *Proteobacteria* [60, 73–75]. T6SS^i^ consists of six subtypes (i1, i2, i3, i4a, i4b, i5). The T6SS clusters identified in *E. bugandensis* and *E. cloacae* were classified according to the TssB amino acid sequence since this protein alone is adequate for T6SS cluster classification [60]. Clusters were grouped and displayed in subtypes based on experimentally validated TssB proteins from other species in the SecReT6 database (Fig. 3) [59, 60]. Seventy-seven TssB protein sequences were identified in the 31 genomes, 62 of which were present in clusters with ≥6 core structural gene components. The 15 TssB sequences that were not identified within clusters may correspond to remnant genes from degraded or non-functional clusters [29, 60]. The T6SS clusters identified in *E. bugandensis* and in *E. cloacae* belonged to either subtype i2 or i3 (Figs 2 and 3). Each T6SS-positive strain carried an i3 cluster, termed T6SS^i3^, which was referred to as the ‘primary’ cluster since it was highly conserved among strains.

**Fig. 4.**
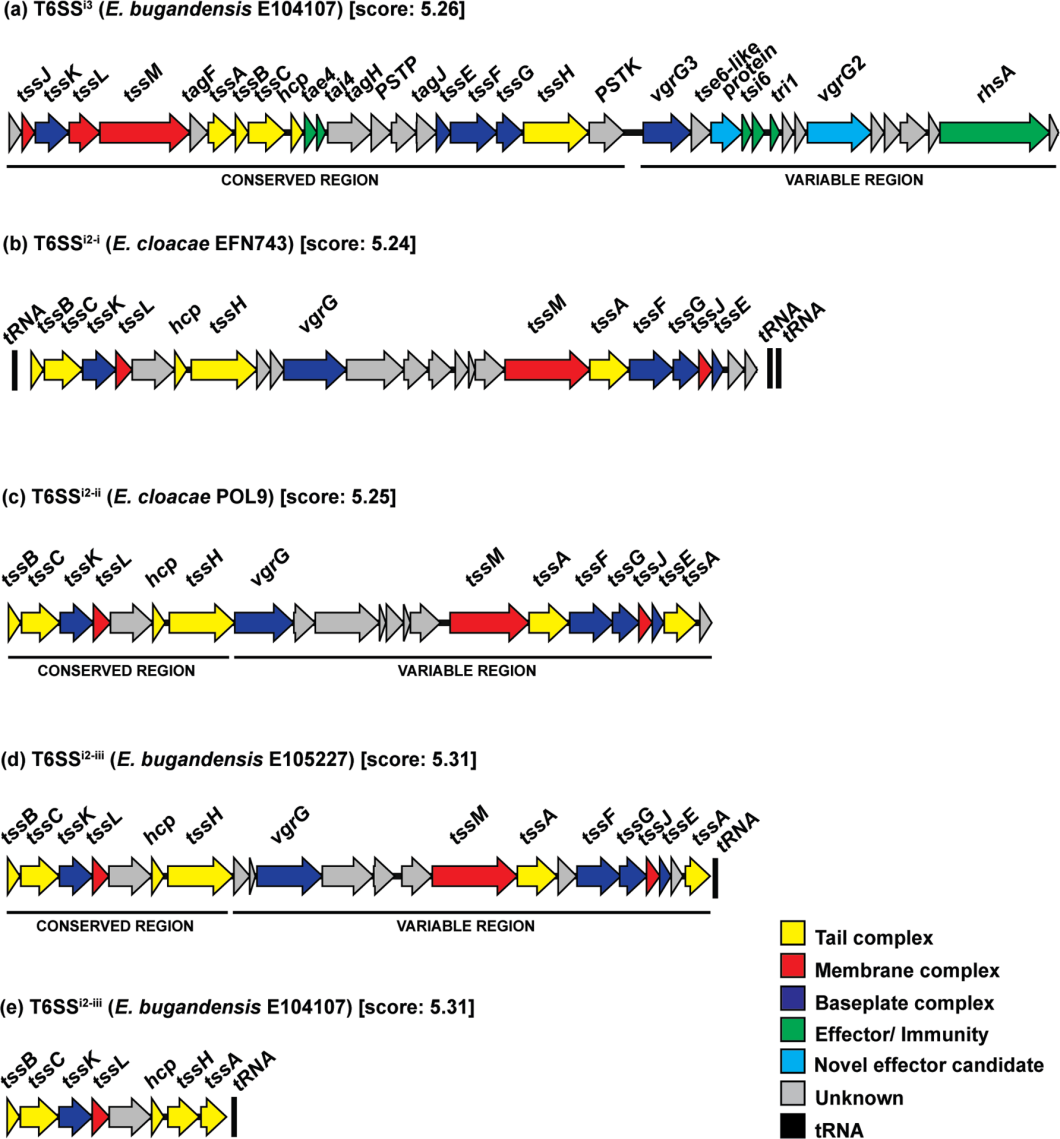
Gene organization of representative T6SS clusters. In all clusters shown, genes encoding components of the T6SS are colour coded as follows: membrane complex (red), baseplate complex (blue), tail complex (yellow) and unknown genes (grey). Flanking tRNAs, if present, are coloured black. Details on all effectors predicted by SecReT6 for all isolates investigated are presented in Table S7. (**a**) *E. bugandensis* E104107 possesses a complete T6SS^i3^ cluster containing all 13 core components required for secretion, along with multiple putative regulators, effectors, and other accessory genes. Known effectors and immunity protein genes are coloured green, and novel effector candidates are coloured cyan. Additional details on the functional analysis of this cluster are given in Table S5. (**b**) Example of a complete T6SS^i2-i^ cluster from *E. cloacae* EFN743. (**c**) Example of a complete T6SS^i2-ii^ cluster from *E. cloacae* POL9. (**d**) Example of a complete T6SS^i2-iii^ cluster from *E. bugandensis* E105227. (**e**) Incomplete T6SS^i2-iii^ cluster in *E. bugandensis* E104107, which also lacks putative effectors. PSTP and PSTK denote genes encoding predicted phosphatase and kinase proteins, respectively. This figure was made using the clinker command within cblaster, and score values indicate cluster similarity. The locus tags corresponding to each gene in this figure are shown in Table S5.

T6SS^i3^ clusters were present in the same genomic location, with every cluster flanked by an upstream *fdhF*/*ydeP* family oxidoreductase gene. Additional genes in the upstream flanking region were also conserved based on visual inspection (Fig. S2), while downstream flanking genes displayed more variability between strains. All T6SS^i3^ clusters contained a highly conserved upstream structural region and a variable downstream ‘*vgrG* island’ encoding many unknown genes (Fig. S2). The upstream structural region encoded almost all structural genes and displayed the same gene organization between strains; this region encoded 12 out of 13 core protein components required for a functional T6SS (TssABCDEFGHJKLM) (Figs 4a and S2). TssI (also known as VgrG) proteins were encoded in the variable downstream region. There was no evidence of gene deletions or duplications, indicating low plasticity in the upstream structural region. The T6SS^i3^ structural region encoded only one conserved Tae4/Tai4 effector/immunity-protein pair [41]. The downstream region of T6SS^i3^ was variable in size and gene organization. Multiple putative effector genes were identified in this region, including genes encoding a Tse6-like protein, an evolved VgrG and RhsA/RhsB proteins. Moreover, T6SS^i3^ encoded predicted immunity proteins with no apparent corresponding effectors. For example, *E. bugandensis* E104107 encoded a Tri1 immunity protein while the gene encoding the corresponding effector, Tre1, was absent in the genome (Fig. 4a). Immunity proteins with no corresponding effectors may provide an advantage against other T6SS-weilding bacteria by increasing protection against their incoming repertoire of effectors, despite not using these effectors themselves. The variable downstream region also encoded several putative regulators, including TagF, TagH, Ser/Thr phosphatases and kinases. Genes encoding various other accessory proteins were also identified, including Eag chaperones, TagJ, and an unknown protein with COG5435 domain and similarity to an Eag chaperone (SciW) which may function like EagT6 to load the Tse6-like protein onto the VgrG spike (Fig. 4a).

Many *E. bugandensis* and *E. cloacae* strains possessed additional clusters belonging to subtype i2. We identified three i2 clusters across the 31 genomes based on genomic location by visual inspection; these were termed T6SS^i2-i^, T6SS^i2-ii^ and T6SS^i2-iii^. The number of i2 clusters was variable across the genomes analysed; strains possessed between zero and two i2 clusters and evidence of deletion was observed. Therefore, we referred to T6SS^i2-i^, T6SS^i2-ii^ and T6SS^i2-iii^ as ‘secondary’ clusters, as they were less conserved in the *E. bugandensis* and *E. cloacae* strains analysed. All *E. bugandensis* strains harboured one T6SS^i2^ cluster, and half of them contained a second T6SS^i2^ cluster. *E. cloacae* strains displayed more variability; most (68.4%) possessed one i2 cluster and only one strain (5.3%) possessed two i2 clusters.

T6SS^i2-i^, T6SS^i2-ii^ and T6SS^i2-iii^ clusters were conserved within the same genomic location in all strains possessing these clusters. Therefore, genomic location was used to differentiate these clusters. T6SS^i2-iii^ clusters were flanked upstream by a gene encoding an EAL domain-containing protein and a downstream *fadJ* gene. Twelve T6SS^i2-iii^ clusters were identified across the 31 genomes, and all non-degraded clusters contained 13 core components required for a functional T6SS (Fig. S3). Unlike T6SS^i3^, which contained structural genes in a block, structural genes in T6SS^i2-iii^ clusters were scattered throughout. However, T6SS^i2-iii^ displayed a conserved gene layout made up of six structural genes in all 12 clusters, including *tssBCKLDH* (Figs 4d and S3). A conserved gene encoding an OmpA domain protein was located between *tssL* and *tssD*; it had predicted structural similarity to TssM and the closest amino acid sequence identity to TssL from *Acidovorax citrulli* AAC00-1. The other TssL protein is a recognizable short form of TssL – also called SciP – which lacks an OmpA extension. The number of unknown genes and *vgrG* genes varied between clusters. Nineteen T6SS^i2-ii^ clusters were identified; they displayed variation in gene layout and organization, differing in orientation, size, and the number of *vgrG* and *tssH* genes (Fig. S4). For example, *E. cloacae* POL9 possessed one *vgrG* gene while *E. bugandensis* FDAARGOS_1496 possessed four *vgrG* genes. T6SS^i2-ii^ clusters were conserved in the same genomic location across all strains that possessed this cluster and were flanked by an upstream OppA peptide ABC transporter substrate-binding protein. The upstream layout of conserved structural genes resembled that of T6SS^i2-iii^, and clusters displayed a conserved layout of *tssBCKLDH* (Figs 4c and S4). Like T6SS^i2-iii^, T6SS^i2-ii^ also possessed a conserved gene encoding an OmpA-like protein located between *tssL* and *tssD* (Fig. 4c). Only two T6SS^i2-i^ clusters were identified in two *E. cloacae* strains within our dataset, revealing that this cluster is not widespread across *E. cloacae* and *E. bugandensis* strains. Both T6SS^i2-i^ clusters were identified by genomic location and were found in the same genomic region, flanked by an upstream FAD-NAD(P) binding protein (Fig. S5). One T6SS^i2-i^ was complete and contained all 13 T6SS core components; however, the other T6SS^i2-i^ cluster was incomplete and lacked several core structural genes required for secretion. Similar to T6SS^i2-ii^ and T6SS^i2-iii^, the complete T6SS^i2-i^ cluster displayed a gene layout consisting of six structural genes, including *tssBCKLDH* (Figs 4b and S5). T6SS^i2-i^ also possessed a conserved gene encoding an OmpA-like protein located between *tssL* and *tssD*.

### Genomic layout of the T6SS in the *E. bugandensis* E104107 model strain reveals two T6SS clusters and multiple auxiliary modules

*E. bugandensis* E104107 is a clinical isolate in our lab collection that was obtained from a respiratory sample. It is highly resistant to polymyxins [76] and virulent in the *Galleria mellonella* infection model (data not shown), as also reported for the *E. bugandensis* EB-247 type strain [54]. For these reasons, we used E104107 as a model *E. bugandensis* strain to explore the biology of infection by this species; in this study, we focused on this strain for further analysis of the T6SS. E104107 harbours two T6SS clusters, a complete primary T6SS^i3^ and an incomplete secondary T6SS^i2-iii^ cluster that displayed evidence of deletion (Fig. 4a, e).

The *E. bugandensis* E104107 incomplete T6SS^i2-iii^ cluster harboured seven (*tssABCDHKL*) of the 13 core T6SS gene components. All components of the tail complex were present; however, genes encoding the baseplate (TssEFG and VgrG) and membrane (TssJM) complexes were absent, suggesting T6SS^i2-iii^ is insufficient for secretion. This cluster was determined as a T6SS^i2-iii^ based on genomic location in comparison to other strains included in the dataset. It is unknown whether this T6SS^i2-iii^ cluster is non-functional or may utilize T6SS^i3^ structural components under certain conditions to form a functional T6SS. T6SS^i2-iii^_E104107_ possesses only structural genes, and no unknown or predicted effectors were identified; its gene organization was identical to the first six genes of T6SS^i2-iii^ from other *E. bugandensis* and *E. cloacae* strains (including *tssBCKLD*). The adjacent *tssH* gene is truncated, and its product was 99.0 % identical to the first 402 aa of T6SS^i2-iii^ TssH from *E. bugandensis* E105227, another strain from our dataset with a complete T6SS^i2-iii^ cluster. Additionally, a tRNA was located immediately downstream of T6SS^i2-iii^, adjacent to *tssA*. tRNAs are often found adjacent to pathogenicity islands and may serve as sites for foreign DNA insertion via horizontal gene transfer, and also as sites for homologous recombination between genomes [77]. Together, these findings indicate a deletion event may have occurred within T6SS^i2-iii^_E104107_, resulting in the loss of several structural and unknown-function genes.

E104107 lacked a T6SS^i2-ii^ cluster; however, *tssBC* remnant genes were identified in the same genomic location as T6SS^i2-ii^ in other strains. There were no putative effectors or other T6SS-associated genes surrounding *tssBC*_E104107_ *,* and TssB_E104107_ had 98.2 % amino acid sequence similarity to T6SS^i2-ii^ TssB of *E. bugandensis* type strain EB-247, an example of an *E. bugandensis* strain with a complete T6SS^i2-ii^ cluster. Additionally, 151 of 153 aa of TssC_E104107_ were identical to the beginning of TssC_EB-247_ in T6SS^i2-ii^, indicating the presence of a truncated T6SS^i2-ii^ *tssC* gene in the E104107 genome. Together, these findings indicate a deletion event occurred in *tssC* that eliminated almost every gene of T6SS^i2-ii^ in *E. bugandensis* E104107. No T6SS-assocated genes were located at the same genomic location as T6SS^i2-i^ in other strains, demonstrating the absence of this cluster in *E. bugandensis* E104107.

T6SS^i2-ii^ is functional in *E. cloacae* ATCC 13047, playing a role in biofilm formation, adherence to epithelial cells and intestinal colonization [40]. However, it only contains 12 of 13 core components, lacking *hcp*. Deletion of T6SS^i2-ii^ *tssH*_ATCC13047_ resulted in reduced adherence to HeLa cell monolayers [40] and reduced mouse intestinal colonization at 3 days post-infection. Moreover, a double deletion mutant of T6SS^i3^ *tssH*_ATCC13047_ and T6SS^i2-ii^ *tssH*_ATCC13047_ resulted in significantly reduced colonization compared to wild-type *E. cloacae* ATCC 13047 [40]. T6SS^i3^_ATCC13047_ was also implicated in bacterial competition; deletion of T6SS^i3^ *tssH*_ATCC13047_ abolished the competitiveness of *E. cloacae* ATCC 13047 during inter-bacterial competition, while deletion of T6SS^i2-ii^ *tssH*_ATCC13047_ had no significant effect. Compared to *E. cloacae* ATCC 13047, there were differences in gene organization of the variable downstream VgrG region of both T6SS^i3^ clusters.

T6SS^i3^_E104107_ encodes all 13 core structural components required for secretion (TssA-M) with the same gene layout as all T6SS^i3^ clusters from the other strains in our dataset. In addition, T6SS^i3^_E104107_ carries genes encoding numerous putative effectors, immunity proteins, regulators and chaperones (Table S5). Moreover, *E. bugandensis* E104107 contains nine *hcp* genes scattered across the genome. One *hcp* gene was found in T6SS^i3^ (*hcp6*) and T6SS^i2-iii^ (*hcp8*). The remaining seven lone *hcp* genes were scattered across the genome, often in association with an array of putative effector genes, representing auxiliary modules.

### A T6SS^i3^ *vgrG* gene encodes a novel putative metallopeptidase domain

**Fig. 5.**
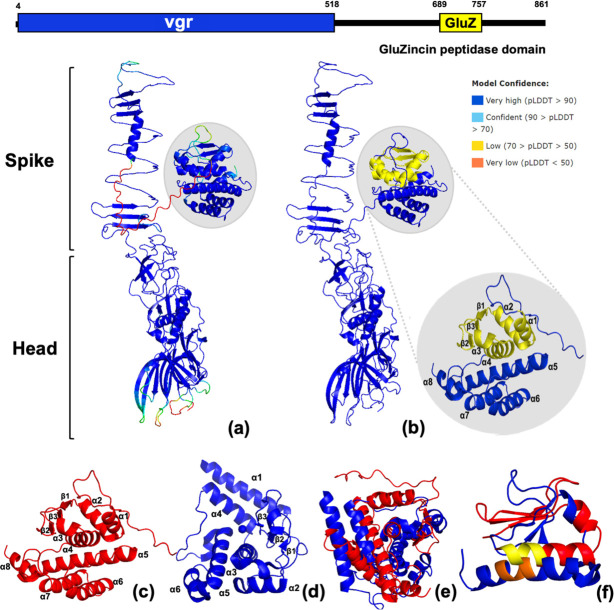
Predicted structure of the VgrG2 monomer and comparison of VgrG2 effector domain with *P. aeruginosa* PAO1 VgrG2b effector domain. (**a**) VgrG2 structure was confidently predicted using Colabfold (Alphafold 2.0 and MMseqs2) and was coloured by pLDDT confidence score. (**b**) VgrG2-predicted structure displays a head and spike portion with a putative effector domain (highlighted by grey circle). The VgrG2 putative effector domain exhibits a recognizable gluzincin-family metallopeptidase domain (yellow) and possesses a total of eight α-helices and three anti-parallel β-sheets to form a compact structure on the side of the VgrG spike. (**c**) The putative effector domain of *E. bugandensis* E104107 (red) was predicted using Colabfold (Alphafold 2.0 and MMseqs2) and (**d**) the crystal structure of *P. aeruginosa* PAO1 VgrG2b effector domain (PDB ID: 6H56) (blue). (**e**) E104107 (red) and PAO1 (blue) effector domain structures could not be confidently aligned using PyMoL, demonstrating that they are distinct proteins. (**f**) Modelling of the putative E104107 effector domain (red, with yellow indicating the conserved HEXXH motif) and PAO1 effector domain active site (blue, with orange indicating the conserved HEXXH motif) reveals similar structures.

Two VgrG proteins were encoded within *E. bugandensis* E104107 T6SS^i3^. The predicted structure of VgrG2 (861 aa) corresponds to a canonical VgrG head, with an extended spike portion at the beginning of the C-terminal region. The C-terminal region has an extension positioned at the side of the spike portion (Fig. 5). This extension, joined to the main canonical VgrG2 structure by a flexible domain, contains eight predicted α-helices and three anti-parallel β-sheets. The C-terminal extension exhibits a gluzincin metallopeptidase domain at amino acid positions 689–757. The gluzincin metallopeptidase domain was identified on the C-terminal extensions of two *E. bugandensis* strains and no *E. cloacae* strains within our curated dataset. The gluzincin family are a group of thermolysin-like peptidases, including several zinc-dependent metallopeptidases with a diverse variety of functions. A similar metallopeptidase domain is present on the C-terminal extension of VgrG2b of *Pseudomonas aeruginosa* PAO1, which elicits toxicity in the periplasm of prey bacteria by causing membrane blebbing and lysis [78]. VgrG2b can also promote bacterial internalization in epithelial cells by interacting with microtubule components [26]. However, the molecular mechanisms behind these phenotypes remain unclear. Structural and sequence comparisons of PAO1 and E104107 C-terminal extensions demonstrated that both are distinct proteins with different structures (Fig. 5c–f). PAO1 VgrG2b had six α-helices and three anti-parallel β-sheets, whereas E104107 VgrG2 possessed eight α-helices and three anti-parallel β-sheets. The full PAO1 and E104107 putative effector domain alone, as well as the recognizable metallopeptidase domain alone, did not complementarily align, underlining the distinctness of these proteins (Fig. 5e). PAO1 VgrG2b has a zinc-binding motif, HEXXH, as part of the active site, which was also present in the predicted gluzincin peptidase domain of the E104107 VgrG2 C-terminal extension. Structural alignment of this binding domain and surrounding amino acids revealed a similar arrangement of two α-helices and three β-sheets surrounding the zinc-binding domain within each metallopeptidase fold (Fig. 5f).

**Fig. 6.**
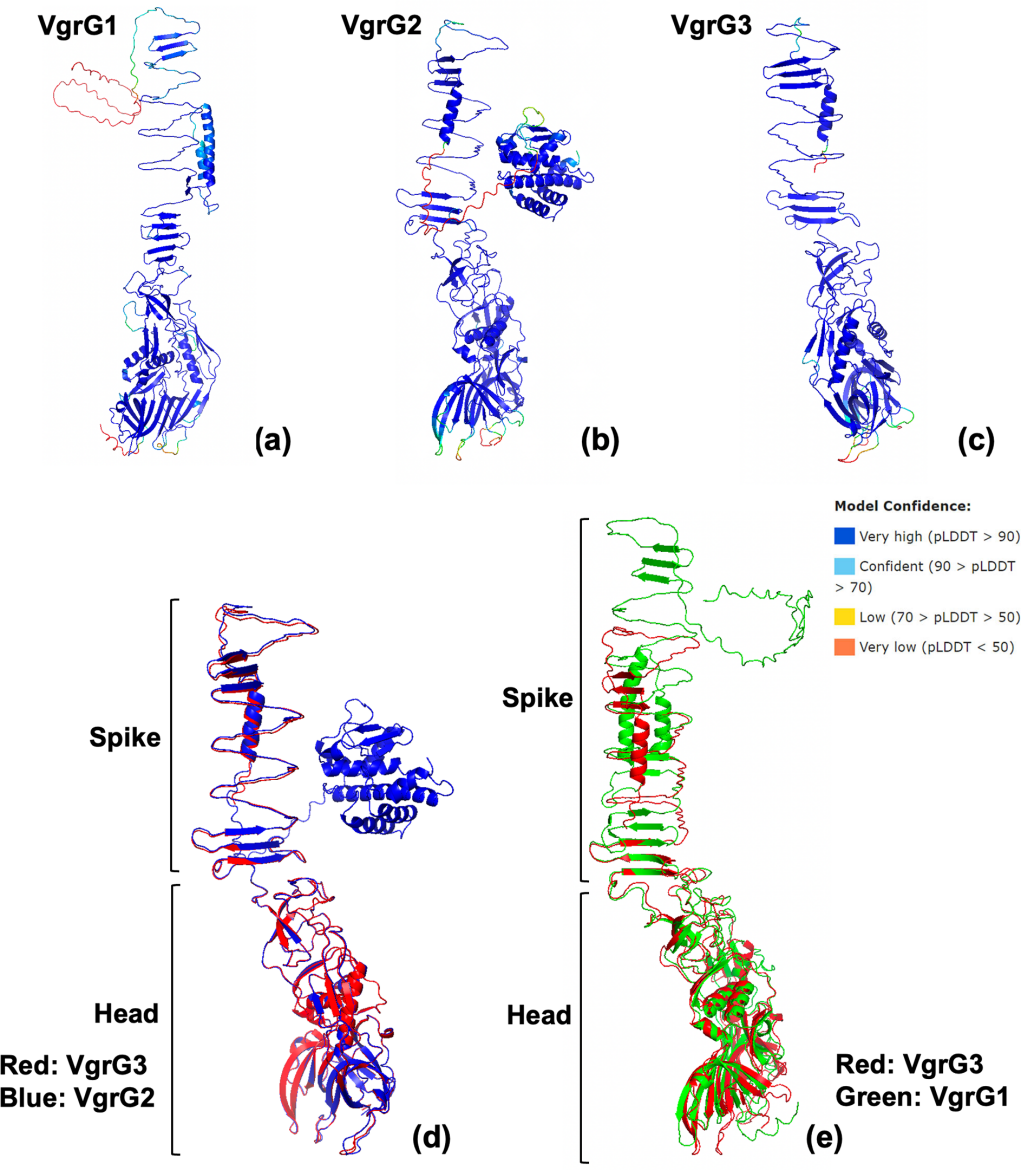
Comparison of three VgrG structures located in the *E. bugandensis* E104107 genome. (**a–c**) Structures of three VgrG proteins located in the *E. bugandensis* E104107 genome were confidently modelled using Colabfold (Alphafold 2.0 and MMseqs2) and are coloured by pLDDT score. (**d**) Structural alignment using PyMoL reveals that the head and spike portion of VgrG2 and VgrG3 are identical, and VgrG2 possesses an additional, putative, C-terminal effector domain located on the side of the spike. (**e**) Structural alignment reveals that VgrG1 and VgrG3 possess a highly similar head portion while the spike portions are different. The VgrG1 spike portion extends further than VgrG3 to elongate the structure.

VgrG3 had an identical structure to the first ~645 aa of VgrG2, representing the head and spike portions (Fig. 6d). Unlike VgrG2, VgrG3 lacked predicted effector domains on the C-terminal extension. Therefore, VgrG3 is unlikely to be an evolved effector, and the C-terminal extension may function to sharpen the VgrG spike for more efficient puncturing and effector delivery. VgrG3 was identified in two *E. bugandensis* strains; however, VgrG homologues lacking predicated effector domains on their C-terminal regions were also identified. Furthermore, 17 *E. cloacae* strains possessed homologues with 93–94 % amino acid sequence identity to VgrG3. The VgrG homologues differed by a recognizable baseplate-subunit-and-tail-lysozyme domain at amino acid positions 493–564.

### Two putative anti-bacterial PAAR proteins are located within T6SS^i3^

We searched the *E. bugandensis* E104107 genome for the presence of PAAR-domain containing genes. All identified PAAR domain-containing genes possessed additional motifs which may represent toxins. No genes encoding exclusively PAAR domains were identified. T6SS^i3^ encodes two PAAR proteins, RhsA and a Tse6-like protein. PAAR (Proline–Alanine–Alanine–aRginine) proteins assemble onto the blunt end of the VgrG spike as a sharp, conical extension to facilitate penetration of target cell membranes [79]. The PAAR domain may also act as a chaperone and often exhibits C-terminal extensions which encode effectors [52, 79–81]. RhsA and RhsB proteins are crucial for bacterial killing and efficient Hcp secretion in *E. cloacae* ATCC 13047 [52]. Bacteria can secrete Rhs effectors to antagonize both bacteria and eukaryotic cells; however, not all are secreted in a T6SS-dependent manner [82–85]. Rhs proteins contain Rhs/YD-peptide repeats which form a β-cage structure to encapsulate the C-terminal toxin domain and may prevent autointoxication [52]. *E. cloacae* ATCC 13047 requires Rhs PAAR motifs to stabilize the VgrG trimer and the β-cage for efficient T6SSi3 assembly [52]. These domains ensure cargo effectors are loaded onto VgrG prior to T6SS assembly. The function of the RhsA C-terminal toxin domain is unknown. However, autoproteolysis releases the C-terminal toxin domain from RhsA and this cleavage is required for anti-bacterial activity [52]. The RhsB C-terminal domain contains a Tox-GHH DNase domain separated from the Rhs/YD core by a DPxGL motif [52]. Similarly, this motif separates the Rhs/YD core and C-terminal toxin domain of RhsA. RhsB is absent in the *E. bugandensis* E104107 genome, but RhsA was identified in T6SS^i3^ and exhibited 87.3 % amino acid sequence identity to ATCC 13047 RhsA. The PAAR and Rhs/YD regions of E104107 and ATCC 13047 RhsA had high sequence identity, but the C-terminal domains were different. E104107 contained a recognizable domain of unknown function (DUF3990). The function of this domain, which is also found in T6SS-associated Rhs proteins from other bacteria [82], is unknown. In ATCC 13047, RhsA can intoxicate bacteria but its function in *E. bugandensis* remains to be established. The *rhsA* gene was conserved across the 31 genomes and was found in all *E. bugandensis* and 12 (63.2 %) *E. cloacae* strains analysed, while *rhsB* was highly conserved in *E. cloacae* and was identified in 18 strains (94.7%); however, *rhsB* was not conserved in *E. bugandensis* as it was only identified in one strain.

**Fig. 7.**
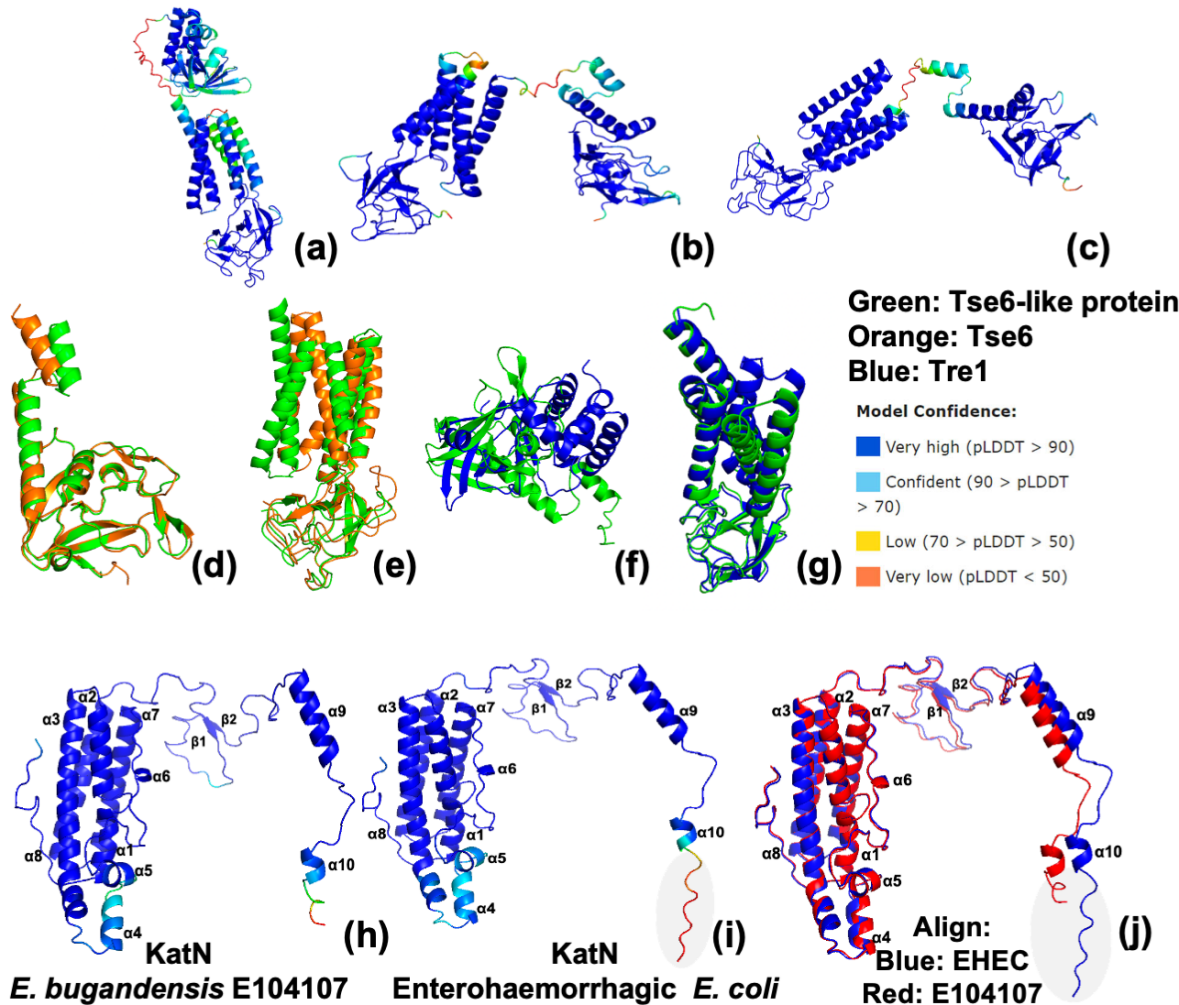
Comparison of *E. bugandensis* E104107 Tse6-like protein structure with Tse6 from *P. aeruginosa* and Tre1 from *S. proteamaculans,* and comparison of *E. bugandensis* E104107 and enterohaemorrhagic *Escherichia coli* (EHEC) KatN structures. Structures of (**a**) Tre1 from *S. proteamaculans *, (**b**) Tse6-like protein from *E. bugandensis* E104107 and (**c**) Tse6 from *P. aeruginosa* were confidently modelled using Colabfold (Alphafold 2.0 and MMseqs2) and coloured by pLDDT score. Complete predicted structures of Tse6 and Tre1 were used since only the toxin domains have been crystallized. (**d**) Alignment of E104107 Tse6-like protein toxin domain (green) and *P. aeruginosa* Tse6 toxin domain (orange) structures display high structural similarity. (**e**) Alignment of E104107 Tse6-like protein PAAR+ transmembrane regions (green) and *P. aeruginosa* Tse6 PAAR+ transmembrane regions (orange) display lower structural similarity. (**f**) Structures of the E104107 Tse6-like protein toxin domain (green) and Tre1-toxin domain (blue) were aligned and exhibit different structures. (**g**) Alignment of the E104107 Tse6-like protein PAAR+ transmembrane regions (green) and Tre1 PAAR+ transmembrane regions (blue) display high structural similarity. (**h**) The structure of *E. bugandensis* KatN and (**i**) EHEC KatN were confidently predicted using Colabfold (Alphafold 2.0 and MMseqs2) and are coloured by pLDDT score. (**j**) Structural alignment using PyMoL reveals that E104107 KatN (red) and EHEC KatN (blue) are highly similar.

E104107 T6SS^i3^ also contains a Tse6-like PAAR protein. Tse6 is a NAD(P)+ glycohydrolase effector in *P. aeruginosa* that degrades NAD+ and NADP+, both essential dinucleotides [86]. An EagT6 chaperone protects the hydrophobic transmembrane regions of Tse6 from aggregation and degradation prior to loading onto VgrG [81]. Tse6 is believed to be delivered by the T6SS into the periplasm of target cells where it spontaneously embeds into the membrane and allows the toxin domain to translocate across the inner membrane into the cytoplasm to exert its toxicity [81]. Tse6 also requires the essential housekeeping protein, translation elongation factor Tu (EF-Tu), for entry into target cells; it is unknown how EF-Tu facilitates entry [81, 86]. E104107 Tse6-like protein had 36.9 % amino acid sequence identity to Tse6. The transmembrane regions of *P. aeruginosa* Tse6 did not align with the transmembrane regions of *E. bugandensis* E104107 Tse6-like protein, but the Ntox46 toxin domain of the E104107 Tse6-like protein aligned identically with the Ntox46 domain of Tse6 from *P. aeruginosa* (Fig. 7d–e). E104107 Tse6-like protein also possessed 66.5 % amino acid sequence identity to Tre1 from *Serratia proteamaculans *. Although the toxin domains of Tre1 and E104107 Tse6-like protein were distinct, the N-terminal PAAR and transmembrane helices of the Tse6-like protein displayed high structural similarity to Tre1 (Fig. 7f–g). Tre1 is an ADP-ribosyltransferase that blocks cell division by modifying the essential bacterial tubulin-like protein, FtsZ [87]. Tse6-like protein was less conserved across the 31 genomes and was identified in two *E. bugandensis* and two *E. cloacae* strains; these findings highlight the diversity of effectors encoded in *E. cloacae* and *E. bugandensis* strains and support the hypothesis that Tse6-like protein may have evolved from a fusion event between two effectors. E104107 T6SS^i3^ contains both Tsi6 and Tri1, the immunity proteins for Tse6 and Tre1, respectively. Tri1 protects from self-intoxication by two modes, including active site occlusion and removing enzymatic modification of proteins by its corresponding effector, Tre1 [87]. T6SS^i3^ possesses an adjacent additional predicted ADP-ribosyl glycohydrolase immunity protein. Both immunity proteins contain recognizable ADP-ribosyl glycohydrolase domains and have significant sequence similarity to Tri1 from *S. proteamaculans *. E104107 Tri1 displayed 75 % amino acid sequence identity to *S. proteamaculans* Tri1, and the adjacent additional ADP-ribosyl glycohydrolase displayed 50 % sequence identity. No corresponding effectors were identified for these immunity proteins, but it is unknown whether effectors are located elsewhere in the genome. Alternatively, rather than protecting against self-intoxication by effectors, these lone immunity proteins may have been acquired by *E. bugandensis* E104107 to increase immunity against attacking cells.

### T6SS^i3^ harbours a Tae4-like anti-bacterial effector

Tae4 is the only effector encoded within the structural region of E104107 T6SS^i3^. Tae4 (Type VI Amidase Effector 4) is an anti-bacterial amidase effector with a N1pC/P60 domain [41]. It acts as a dl-endopeptidase that hydrolyses the peptide bond between γ-d-glutamic and meso-diaminopimelic acid [41]. The *tae4* gene is highly conserved across the 31 genomes of our dataset; all *E. bugandensis* and 14 *E. cloacae* (73.7%) strains carry a *tae4* gene. The immunity protein, Tai4 (Type VI Amidase Immunity 4), protects against self-intoxication by forming a dimer with Tae4. The Tae4/Tai4 interface comprises a heterotetramer of two Tae4 molecules and a Tai4 dimer in solution [41]. Inhibition requires a Tai4 dimer as one subunit is important for binding Tae4 while the other blocks Tae4 activity [41]. E104107 Tae4 displayed 96.3 % amino acid sequence identity to *E. cloacae* ATCC 13047 Tae4, while E104107 Tai4 had 79.5 % sequence identity to ATCC 13047 Tai4.

### Additional putative T6SS effectors are present outside T6SS clusters

A third *vgrG* gene (*vgrG1*) was identified outside of the T6SS clusters and was not associated with any other T6SS genes. The head portion of VgrG1 was structurally identical to that of VgrG2 and VgrG3 (Fig. 6e). However, there were differences in the spike portion; the VgrG1 spike was longer and consisted of additional anti-parallel β-sheets and α-helices. Additionally, VgrG1 displayed a recognizable DUF2345 domain on its C-terminal extension. VgrG1 was identified in two *E. bugandensis* strains within our curated dataset; however, VgrG homologues also possessing a DUF2345 domain were identified in *E. bugandensis* and *E. cloacae* strains. E104107 VgrG1 was associated with an adjacent putative effector displaying DUF3289 and M23 peptidase domains. M23 family metallopeptidases are zinc-dependent and degrade peptidoglycan [88]. The function of DUF3289 is unknown but has been identified in T6SS genes, including PAAR-related proteins [89]. A small, unknown ORF was located immediately downstream of the putative peptidase effector and may represent the corresponding immunity protein. This M23 family peptidase has not been explored and is not present in the SecReT6 database of known effectors, suggesting it is a novel effector. The M23 peptidase gene was less conserved across the 31 genomes and was only identified in two *E. bugandensis* strains, highlighting the diversity of encoded effectors in *E. bugandensis* and *E. cloacae *.

Another gene, also not associated with T6SS clusters, was identified in the *E. bugandensis* E104107 genome, and encoded a manganese-containing catalase with 89 % amino acid sequence identity to KatN from enterohemorrhagic *Escherichia coli* (EHEC). The predicted structures of EHEC and E104107 KatN were almost identical (Fig. 7h–j). Both KatN structures displayed 10 α-helices and two β-sheets with short regions at the C-termini that could not be accurately predicted by alphafold. All 31 strains possessed *katN*, revealing that it appears highly conserved across *E. bugandensis* and *E. cloacae* strains.

Three lone PAAR domain-carrying genes were identified across the *E. bugandensis* E104107 genome. These genes encode N-terminal PAAR domains and C-terminal extensions harbouring S-type pyocin domains, and were termed *ppp1*, *ppp2* and *ppp3* (PAAR Pyocin-S domain-containing Protein). Pyocins are colicin-like, protease-sensitive bacteriocins produced by *Pseudomonas* species; their killing activity is mediated by DNase, lipase, tRNase and channel-forming activities [90]. All proteins were similar in size (521, 565 and 579 aa) and possessed a similarly sized S-type pyocin domain (132–142 aa) at approximately the same amino acid positions (~298–442). Amino acid sequence analysis revealed that Ppp1 and Ppp2 were similar and displayed 66.7 % sequence identity, while Ppp3 displayed only 39.7 % sequence identity to Ppp1 and 32 % sequence identity to Ppp2. Ppp2 carries an additional ‘cytotoxic’ domain (Pfam ID: PF09000) which causes nucleolytic breaks in 16S rRNA, and is found in *Escherichia coli* ribonuclease colicin E3 [91]. These proteins were absent in the SecReT6 database of known effectors. S-type pyocin domains have been identified on the C-terminal extensions of *Enterobacteriaceae hcp* genes and were found to elicit anti-bacterial effects [92]. Therefore, Ppp1–3 may represent novel anti-bacterial effectors. Twenty pyocin-S domain-containing proteins were identified in *E. bugandensis* strains, and 24 were identified in *E. cloacae* strains from our curated dataset.

## Discussion

We have characterized *in silico* the distribution and diversity of T6SS gene clusters in isolates from *E. cloacae* and *E. bugandensis *, both of which are clinically relevant species of the genus *Enterobacter *. From the initial dataset of 90 complete genomes identified as ‘* E. cloacae *’ and ‘* E. bugandensis *’, we demonstrated that only 31 were taxonomically assigned to these species. This highlights the importance of verifying *Enterobacter* strain taxonomy before carrying out comparative studies given the current complexity of *Enterobacter* taxonomy. The genus *Enterobacter* consists of multiple species and the entries in NCBI do not reflect the updated taxonomic classifications [93]. The pipeline outlined in this study can be extended to include more species of the *E. cloacae* complex and other T6SS-weilding bacteria.

The *E. cloacae* and *E. bugandensis* strains in our curated dataset encode up to three T6SS clusters, of which T6SS^i3^ is present and complete in most strains. The organization of T6SS^i3^ structural genes was also conserved across all strains; however, strains exhibited diversity in the array of encoded effectors and unknown genes. Many strains harboured one or two additional clusters belonging to subtype i2. We identified three i2 clusters across the 31 strains (termed T6SS^i2-i^, T6SS^i2-ii^ and T6SS^i2-iii^), and these were conserved in genomic location but displayed evidence of deletion and diversity in gene organization. T6SS cluster diversity may have resulted from the arms-race between bacteria to outcompete one another in their environmental niche. Furthermore, gain and loss of clusters may be related to fitness costs, balancing the high energy cost associated with T6SS maintenance, resulting in deletion events, and the specific pressures in the niches where these isolates evolved.

Detailed analysis of our model isolate, *E. bugandensis* E104107, revealed nine *hcp* genes. While T6SS^i3^_E104107_ and T6SS^i2-iii^_E104107_ clusters contained one *hcp* gene each, the other seven *hcp* genes were scattered across the genome. They were often associated with an array of putative effector genes, representing predicted auxiliary modules. These observations indicate a potential for additional effectors being exchanged in nature among *Enterobacter* strains, or strains from other species, which may be significant for niche adaption. Similar auxiliary modules were also present in the other 30 genomes included in our study; work is ongoing to characterize the distribution and diversity of effectors within these modules. Furthermore, we identified several previously described and unknown putative effectors in *E. bugandensis* E104107, including two VgrG, three pyocin-S domain-containing PAAR proteins (Ppp1–3), Tse6-like protein, KatN, Tae4, RhsA and peptidase proteins. Pyocins may have been acquired by *Enterobacter* species by horizontal gene transfer and fused with PAAR proteins to produce new anti-bacterial effectors. While the T6SS may deliver effectors to the cytoplasm or periplasm of target cells [81, 94], it is also possible that the pyocin domain may aid in delivering effectors from the periplasm to the cytoplasm of target cells. In EHEC, KatN expression was induced by the stress regulators, RpoS and OxyR, contributing to virulence by promoting intracellular survival within macrophages [95]. These findings suggest KatN could have a similar function in *E. bugandensis *. VgrG1 has a DUF2345 domain, but the function of this domain in *Enterobacter* species is unknown. In *Escherichia coli *, DUF2345 was shown to stabilize the interaction between VgrG and the phospholipase effector, Tle1 [20]. However, a single amino acid change in *Acinetobacter baumannii* VgrGi DUF2345 was observed to inhibit T6SS function, possibly by eliminating the energy cost associated with producing functional T6SS since it appears to be unnecessary for *A. baumannii* virulence [96]. Additionally, *Klebsiella pneumoniae* VgrG4 possesses a C-terminal DUF2345 which can intoxicate bacteria and yeast via induction of reactive oxygen species [97] and can serve as an anti-eukaryotic effector promoting fragmentation of the mitochondrial network and activation of innate immune receptor NLRX1 [98].

In summary, our results highlight new anti-eukaryotic and anti-bacterial effector candidates which may facilitate adaption and dominance of *Enterobacter* species within their environmental niches. Notably, the identification of effector candidates, such as KatN and VgrG1 with a potential role in pathogenesis, may provide new avenues to better understand the infection biology of *Enterobacter* species.

## Supplementary Data

Supplementary material 1
