# Supplementary material for: Distribution and diversity of type VI secretion system clusters in Enterobacter bugandensis and Enterobacter cloacae
Source: Microb Genom. 2023 Dec 6;9(12):001148. doi: 10.1099/mgen.0.001148 (PMC10763514; doi:10.1099/mgen.0.001148)
Supplement: Supplementary material 1 [file mgen-9-1148-s001.pdf]

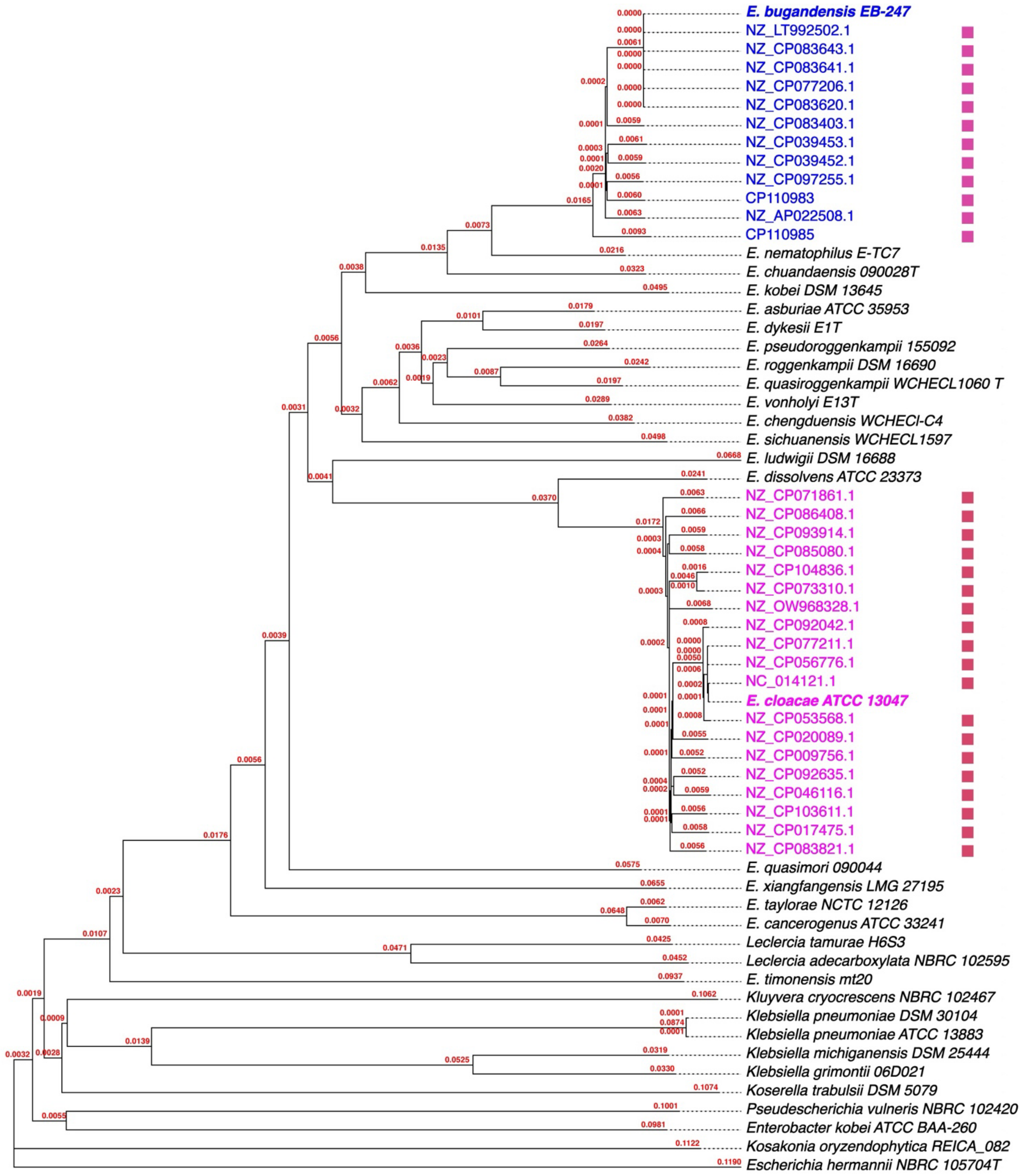

Figure S1. Phylogenetic tree of *E. cloacae* and *E. bugandensis* strains. The correct taxonomy of all 31 *E. cloacae* and *E. bugandensis* strains included in this study was carried out using TYGS with default settings. A phylogenetic tree displaying the relatedness of strains was constructed; *E. cloacae* strains included in this study are highlighted in pink and *E. bugandensis* strains are highlighted in blue.

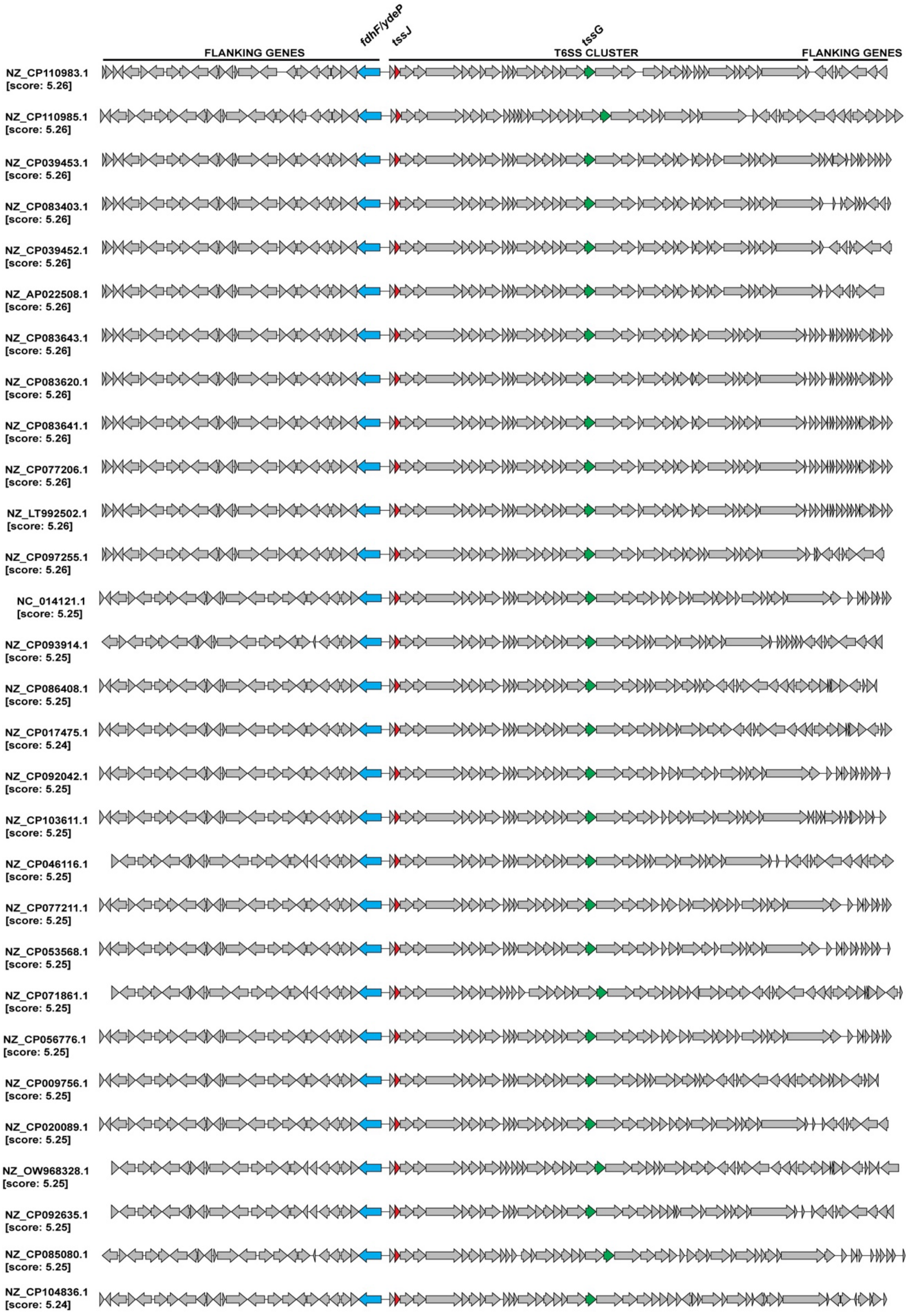

Figure S2. Gene layout of all 29 T6SSi3 clusters identified from *E. bugandensis* and *E. cloacae* strains.

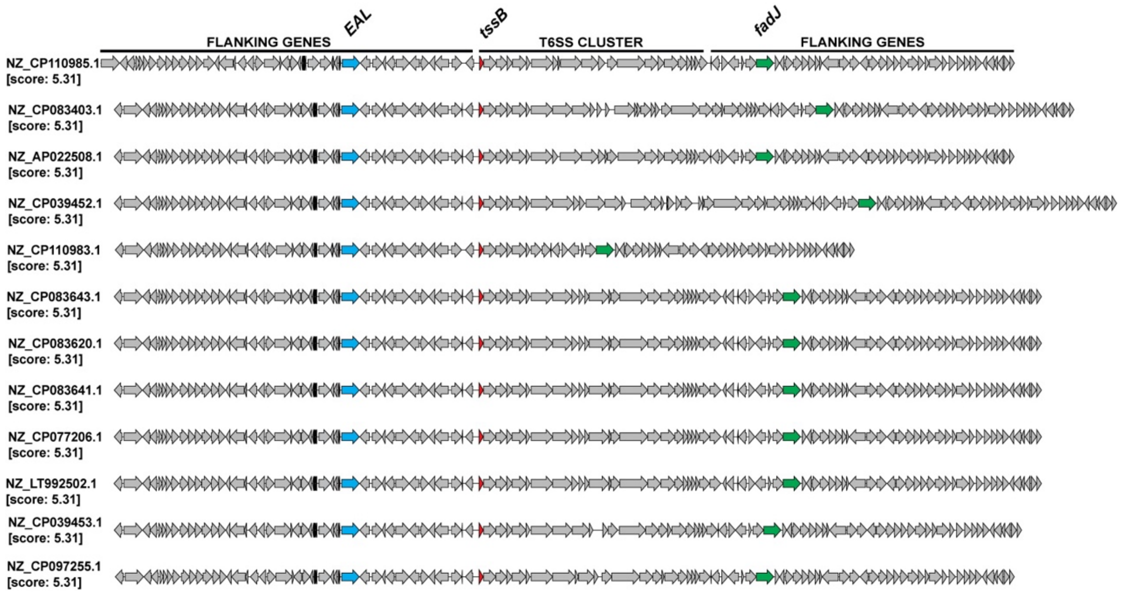

Figure S3. Gene layout of all 12 T6SSi2-iii clusters identified from *E. bugandensis* and *E. cloacae* strains.

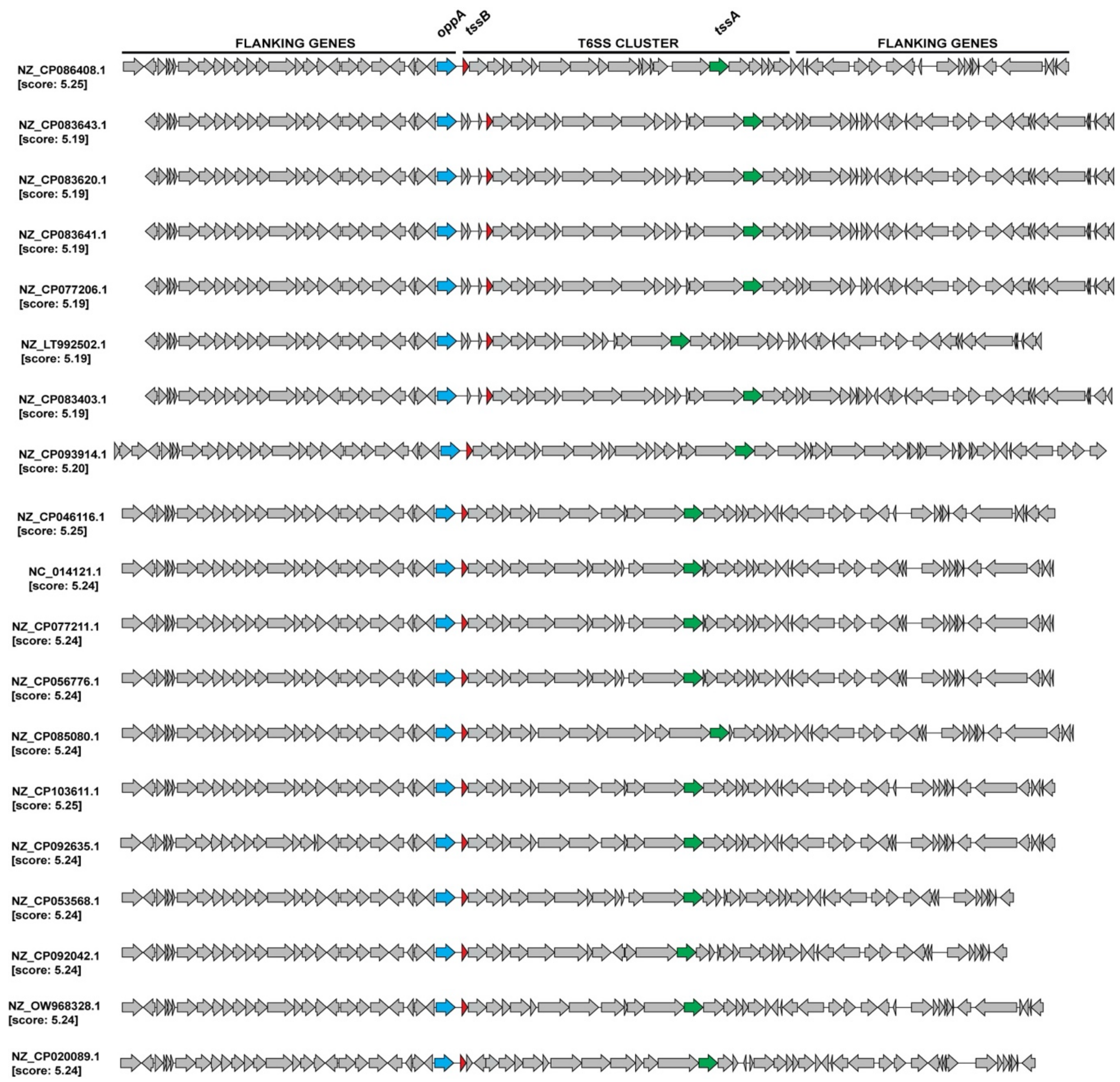

Figure S4. Gene layout all 19 T6SSi2-ii clusters identified from *E. bugandensis* and *E. cloacae* strains.

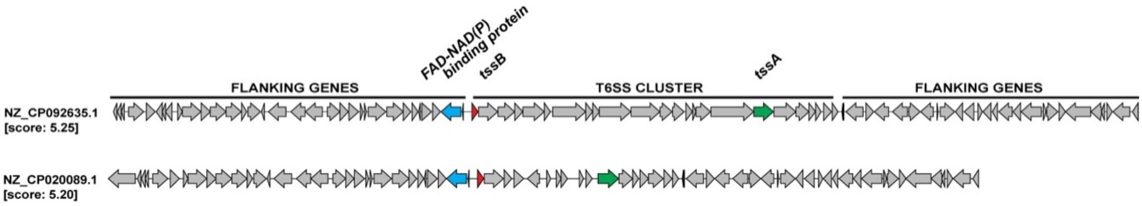

Figure S5. Gene layout of both T6SSi2-i clusters identified from *E. bugandensis* and *E. cloacae* strains.

Table S1. Code used for T6SS analysis

Description	Code
Database creation	$ cblaster makedb [genomesfolder]/*.gff3 -n [databasename]
Search local database	$ cblaster search -m local -db [databasepath].dmnd -qf [queryfilepath].fasta -md 30000 -g 50000 -ig -o [output].fasta -s [session].json
Cluster extraction	$ cblaster extract_clusters [session].json -f genbank
Plot clusters	$ cblaster plot_clusters [session].json
HMMER domain search	$ cblaster search -m hmm -qp PF00000 -db [databasepath].fasta -pfam [pfamfolder]
BlastP database construction	$ makeblastdb -in genomes.fsa -dbtype prot -title genomes -out genomes
BlastP search	$ blastp -db [databasename] -query [queryfilepath] -out [output]

Table S2. Query sequences used to search local databases for T6SS clusters

Cluster	Query sequence name	Query sequence accession number
T6SSi3	FdhF/YdeP family oxidoreductase	WP_309264159.1
	TssJ	WP_309264158.1
	TssG	WP_047367780.1
T6SSi2-i	FAD-NAD(P) binding protein	WP_040019836.1
	TssB	WP_058651512.1
	TssA	WP_040023009.1
T6SSi2-ii	OppA peptide ABC transporter substrate-binding protein	WP_128311290.1
	TssB	WP_013096428.1
	TssA	WP_128310330.1
T6SSi2-iii	EAL-domain containing protein	WP_045631031.1
	TssB	WP_041908834.1
	FadJ	WP_193030413.1

Table S3. T6SS core components used for cluster classification

T6SS core component	Homologues	COG classification	Function
TssA	*impA/vasJ*	COG3515	Tail complex (capping)
TssB	*impB, vipA*	COG3516	Tail complex (sheath)
TssC	*impC, vipB*	COG3517	Tail complex (sheath)
TssD	*hcp*	COG3157	Tail complex (inner tube)
TssE	*mpF, vasS*	COG3518	Baseplate complex
TssF	*impG, vasA*	COG3519	Baseplate complex
TssG	*impH, vasB*	COG3520	Baseplate complex
TssH	*clpV/vasG*	COG0542	Recycling TssB/C sheath
TssI	*vgrG*	COG3501	Baseplate complex
TssJ	*vasD, lip*	COG3521	Membrane complex
TssK	*impJ, vasE*	COG3522	Baseplate complex
TssL	*ompA/dotU*	COG3455	Membrane complex
TssM	*vasK, icmF*	COG3523	Membrane complex

Table S4. Number and subtype classification of T6SS clusters from *E. cloacae* and *E. bugandensis* strains.

Species	Strain ID	Cluster 1	Cluster 2	Custer 3	Number of clusters
E. bugandensis	E104107	i3	i2	-	2
	E105227	i3	i2	-	2
	220	i3	i2	-	2
	1367	i3	i2	-	2
	STN0717_56	i3	i2	-	2
	FDAARGOS_1427	i3	i2	i2	3
	XL95	i3	i2	i2	3
	FDAARGOS_1520	i3	i2	i2	3
	FDAARGOS_1497	i3	i2	i2	3
	FDAARGOS_1496	i3	i2	i2	3
	CMCC(B)45301	i3	i2	-	2
	EB-247	i3	i2	i2	3
E. cloacae	1382	i3	i2	-	2
	ATCC 13047	i3	i2	-	2
	GGT036	i3	-	-	1
	M12X01451	i3	-	-	1
	PIMB10EC27	i3	i2	-	2
	CBG15936	i3	i2	-	2
	DSM 30054	i3	i2	-	2
	CZ862	-	-	-	0
	FDAARGOS 1431	i3	i2	-	2
	ECI_1.VN	i3	i2	-	2
	POL9	i3	i2	-	2
	MY490	i3	i2	-	2
	GX1Z-1L	i3	-	-	1
	AVS0889	i3	i2	-	2
	12961-yvys	-	-	-	0
	SD21	i3	i2	-	2
	EFN743	i3	i2	i2	3
	3143	i3	i2	-	2
	2022CK-00409	i3	i2	-	2

Table S5. Summary of genes within representative *E. bugandensis* and *E. cloacae* T6SS clusters, as depicted in Figure 4 of the main text.

Strain and cluster	Locus Tag	Gene Name	Description of predicted function
E. bugandensis E104107 T6SSi3	OQ482_RS13010	Unknown lipoprotein	Unknown lipoprotein with structural similarity to several peptidases
	OQ482_RS13005	TssJ	Membrane complex
	OQ482_RS13000	TssK	Baseplate complex
	OQ482_RS12995	TssL	Membrane complex
	OQ482_RS12990	TssM	Membrane complex
	OQ482_RS12985	TagF	Putative repressor
	OQ482_RS12980	TssA	Capping protein that co-ordinates T6SS assembly
	OQ482_RS12975	TssB	Contractile sheath subunit. Contracts to propel the puncturing device
	OQ482_RS12970	TssC	Contractile sheath subunit. Contracts to propel the puncturing device
	OQ482_RS12965	Hcp (TssD)	Inner tube of tail complex. Secreted component of the puncturing device
	OQ482_RS12960	Tae4	Known anti-bacterial effector that degrades peptidoglycan
	OQ482_RS12955	Tai4	Immunity protein for Tae4 anti-bacterial effector
	OQ482_RS12950	TagH	Putative activator
	OQ482_RS12945	Ser/Thr phosphatase (PSTP)	Putative TPP-dependent repressor
	OQ482_RS12940	Unknown	Unknown
	OQ482_RS12935	TagJ	Putative accessory protein involved in sheath disassembly
	OQ482_RS12930	TssE	Baseplate complex
	OQ482_RS12925	TssF	Baseplate complex
	OQ482_RS12920	TssG	Baseplate complex
	OQ482_RS12915	TssH	AAA+ ATPase. Recycles TssB/C sheath components
	OQ482_RS12910	Ser/Thr kinase (PSTK)	Putative T6SS activator
	OQ482_RS12900	VgrG3	Secreted component of the puncturing device
	OQ482_RS12895	Unknown	Putative chaperone for Tse6-like protein.
	OQ482_RS12890	Tse6-like protein	PAAR protein with Ntox46 domain, demonstrated as anti-bacterial NAD(P)+ glycohydrolase effector. Novel effector candidate
	OQ482_RS12885	Tsi6	Putative immunity protein for Tse6 anti-bacterial effector
	?	ADP-ribosylglycohydrolase	Putative immunity protein with similarity to Tri1. There has been an error in the NCBI annotation where this gene and *tri1* (below) have been merged into one large gene with multiple stop codons.
	OQ482_RS12880	Tri1	ADP-ribosylglycohydrolase immunity protein for Tre1 anti-bacterial effector
	OQ482_RS12875	Unknown TM protein	Unknown; protein with 4 transmembrane regions
	OQ482_RS12870	Unknown TM protein	Unknown; protein with 4 transmembrane regions
	OQ482_RS12865	VgrG2	Secreted component of the puncturing device. Carries gluzincin metallopeptidase domain. Novel effector candidate

	OQ482_RS12860	Unknown	Unknown
	OQ482_RS12855	Unknown	Unknown
	OQ482_RS12850	Unknown	Unknown
	OQ482_RS12845	EAG chaperone	EAG chaperone displaying DUF1795 domain. May be involved in chaperoning RhsA
	OQ482_RS12840	RhsA	Known anti-bacterial effector with unknown mechanism of action
	OQ482_RS12835	Unknown	Putative immunity protein for RhsA effector
E. cloacae EFN743 T6SSi2-i	MKZ88_RS14670	TssB	Contractile sheath subunit. Contracts to propel the puncturing device
	MKZ88_RS14665	TssC	Contractile sheath subunit. Contracts to propel the puncturing device
	MKZ88_RS14660	TssK	Baseplate complex
	MKZ88_RS14655	TssL	Membrane complex
	MKZ88_RS14650	OmpA family protein	Protein encoding a C-terminal OmpA domain
	MKZ88_RS14645	Hcp (TssD)	Inner tube of tail complex. Secreted component of the puncturing device
	MKZ88_RS14640	TssH	AAA+ ATPase. Recycles TssB/C sheath components
	MKZ88_RS14635	Unknown	Unknown
	MKZ88_RS14630	Unknown	Unknown
	MKZ88_RS14625	VgrG	Secreted component of the puncturing device
	MKZ88_RS14620	Unknown	Unknown
	MKZ88_RS14615	Unknown	Unknown
	MKZ88_RS14610	Unknown	Unknown
	MKZ88_RS14605	Unknown	Unknown
	MKZ88_RS14600	Unknown	Unknown
	MKZ88_RS14595	Unknown	Unknown
	MKZ88_RS14590	TssM	Membrane complex
	MKZ88_RS14585	TssA	Capping protein that co-ordinates T6SS assembly
	MKZ88_RS14580	TssF	Baseplate complex
	MKZ88_RS14575	TssG	Baseplate complex
	MKZ88_RS14570	TssJ	Membrane complex
	MKZ88_RS14565	TssE	Baseplate complex
	MKZ88_RS14560	Unknown	Unknown
	MKZ88_RS14555	Unknown	Unknown
E. cloacae POL9 T6SSi2-ii	LMJ45_RS12350	TssB	Contractile sheath subunit. Contracts to propel the puncturing device
	LMJ45_RS12345	TssC	Contractile sheath subunit. Contracts to propel the puncturing device
	LMJ45_RS12340	TssK	Baseplate complex

	LMJ45_RS12335	TssL	Membrane complex
	LMJ45_RS12330	OmpA family protein	Protein encoding a C-terminal OmpA domain
	LMJ45_RS12325	Hcp (TssD)	Inner tube of tail complex. Secreted component of the puncturing device
	LMJ45_RS12320	TssH	AAA+ ATPase. Recycles TssB/C sheath components
	LMJ45_RS12315	VgrG	Secreted component of the puncturing device
	LMJ45_RS12310	Unknown	Unknown
	LMJ45_RS12305	Unknown	Unknown
	LMJ45_RS12300	Unknown	Unknown
	LMJ45_RS12295	Unknown	Unknown
	LMJ45_RS12290	Unknown	Unknown
	LMJ45_RS12285	Unknown	Unknown
	LMJ45_RS12280	TssM	Membrane complex
	LMJ45_12275	TssA	Capping protein that co-ordinates T6SS assembly
	LMJ45_RS12270	TssF	Baseplate complex
	LMJ45_RS12265	TssG	Baseplate complex
	LMJ45_RS12260	TssJ	Membrane complex
	LMJ45_RS12255	TssE	Baseplate complex
	LMJ45_RS12250	TssA	Capping protein that co-ordinates T6SS assembly
	LMJ45_RS12245	Unknown	Unknown
E. bugandensis E105227 T6SSi2-iii	OQ483_RS16730	TssB	Contractile sheath subunit. Contracts to propel the puncturing device
	OQ483_RS16725	TssC	Contractile sheath subunit. Contracts to propel the puncturing device
	OQ483_RS16720	TssK	Baseplate complex
	OQ483_RS16715	TssL	Membrane complex
	OQ483_RS16710	OmpA family protein	Protein encoding a C-terminal OmpA domain
	OQ483_RS16705	Hcp (TssD)	Inner tube of tail complex. Secreted component of the puncturing device
	OQ483_RS16700	TssH	AAA+ ATPase. Recycles TssB/C sheath components
	OQ483_RS16695	Unknown	Unknown
	?	Unknown	Unknown. NCBI annotation has missed this gene; therefore, it lacks an NCBI locus tag designation.
	OQ483_RS16690	VgrG	Secreted component of the puncturing device
	OQ483_RS16685	Unknown	Unknown
	OQ483_RS16680	Unknown	Unknown
	OQ483_RS16670	Unknown	Unknown

	OQ483_RS16665	TssM	Membrane complex
	OQ483_RS16660	TssA	Capping protein that co-ordinates T6SS assembly
	OQ483_RS16655	Unknown	Unknown
	OQ483_RS16650	TssF	Baseplate complex
	OQ483_RS16645	TssG	Baseplate complex
	OQ483_RS16640	TssJ	Membrane complex
	OQ483_RS16635	TssE	Baseplate complex
	OQ483_RS16630	Unknown	Unknown
	OQ483_RS16625	TssA	Capping protein that co-ordinates T6SS assembly
E. bugandensis E104107 T6SSi2-iii	OQ482_RS16020	TssB	Contractile sheath subunit. Contracts to propel the puncturing device
	OQ482_RS16015	TssC	Contractile sheath subunit. Contracts to propel the puncturing device
	OQ482_RS16010	TssK	Baseplate complex
	OQ482_RS16005	TssL	Membrane complex
	OQ482_RS16000	OmpA family protein	Protein encoding a C-terminal OmpA domain
	OQ482_RS15995	Hcp (TssD)	Inner tube of tail complex. Secreted component of the puncturing device
	OQ482_RS15990	TssH	AAA+ ATPase. Recycles TssB/C sheath components
	OQ482_RS15980	TssA	Capping protein that co-ordinates T6SS assembly

Table S6. List of chromosome and plasmid names and accession numbers for each strain.

Species	Strain	Source	Chromosome accession number	Plasmid name[a]	Plasmid accession number
E. bugandensis	E104107	Clinical, UK	NZ_CP110983.1	pEC104107	NZ_CP110984.1
	E105227	Clinical, UK	NZ_CP110985.1	pEC105227L	NZ_CP110986.1
				pEC105227S	NZ_CP110987.1
	220	Clinical, Germany	NZ_CP039453.1	pSurvcare220	NZ_CP039454.1
	1367	Clinical, Germany	NZ_CP039452.1	-	-
	STN0717-56	Environmental, Japan	NZ_AP022508.1	-	-
	FDAARGOS 1427	Clinical, Germany	NZ_CP077206.1	unnamed1	NZ_CP077207.1
				unnamed2	NZ_CP077208.1
	XL95	Environmental, China	NZ_CP083403.1	unnamed	NZ_CP083404.1
	FDAARGOS_1520	Unknown, Germany	NZ_CP083620.1	unnamed	NZ_CP083621.1
	FDAARGOS_1497	Unknown, Germany	NZ_CP083641.1	unnamed	NZ_CP083642.1
	FDAARGOS_1496	Unknown, Germany	NZ_CP083643.1	unnamed	NZ_CP083644.1
	CMCC(B)45301	Unknown, China	NZ_CP097255.1	pCMCCB45301	NZ_CP097254.1
	EB-247	Clinical, Tanzania	NZ_LT992502.1	-	-
E. cloacae	1382	Clinical, Spain	NZ_OW968328.1	P1	NZ_OW968329.1
				P2	NZ_OW968330.1
	ATCC13047	Clinical, Unknown	NC_014121.1	pECL_A	NC_014107.1
				pECL_B	NC_014108.1
	GGT036	Unknown, South Korea	NZ_CP009756.1	-	-
	M12X01451	Clinical, Unknown	NZ_CP017475.1	pM12X01451	NZ_CP017473.1
	PIMB10EC27	Clinical, Vietnam	NZ_CP020089.1	pEC27-1	NZ_CP020090.1
				pEC27-2	NZ_CP020091.1
	CBG15936	Clinical, China	NZ_CP046116.1	pTEM-CBG	NZ_CP046117.1
				pNDM1-CBG	NZ_CP046118.1
	DSM 30054	Unknown, unknown	NZ_CP056776.1	P1	NZ_CP056777.1
				p2	NZ_CP056778.1
	CZ862	Clinical, Czech Republic	NZ_CP073310.1	pCZ862_1	NZ_CP073311.1
				pCZ862_ColRNAI	NZ_CP073312.1
				pCZ862_GES1_GES5	NZ_CP073313.1
				pCZ862_IncFIB	NZ_CP073314.1
	FDAARGOS 1431	Unknown, Germany	NZ_CP077211.1	unnamed1	NZ_CP077212.1
				unnamed2	NZ_CP077213.1
	ECl_1.VN	Clinical, South Africa	NZ_CP085080.1	-	-
	POL9	Environmental, China	NZ_CP086408.1	pPOL9A	NZ_CP086409.1
				pPOL9B	NZ_CP086410.1
	MY490	Animal, China	NZ_CP053568.1	pMY490-mcr-9	NZ_CP053569.1
				pMY490-2	NZ_CP053570.1
	GX1Z-1L	Animal, China	NZ_CP071861.1	pGX1Z-1L	NZ_CP071862.1
	AVS0889	Environmental, Switzerland	NZ_CP092042.1	pAVS0889-a	NZ_CP092043.1
				pAVS0889-b	NZ_CP092044.1
				pAVS0889-c	NZ_CP092045.1
				pAVS0889-d	NZ_CP092046.1
				pAVS0889-e	NZ_CP092047.1
	12961-yvys	Clinical, China	NZ_CP083821.1	p12961_1	NZ_CP083822.1
				p12961_2	NZ_CP083823.1
	SD21	Clinical, China	NZ_CP093914.1	pSD21_266kb	NZ_CP093915.1
				pSD21_mcr8	NZ_CP093916.1
				pSD21_54kb	NZ_CP093917.1
				pSD21_4kb	NZ_CP093918.1
	EFN743	Clinical, Ghana	NZ_CP092635.1	p743-OXA-181	NZ_CP092636.1
				p2	NZ_CP092637.1
				p3	NZ_CP092638.1
	3143	Clinical, USA	NZ_CP103611.1	pMB6956_1	NZ_CP103612.1
				pMB6956_2	NZ_CP103613.1

	2022CK-00409	Clinical, USA	NZ_CP104836.1	unnamed1	NZ_CP104837.1
				unnamed2	NZ_CP104838.1
				unnamed3	NZ_CP104839.1
				unnamed4	NZ_CP104840.1
				unnamed5	NZ_CP104841.1
				unnamed6	NZ_CP104842.1

[a] -, indicates plasmids not available from NCBI

Table S7. Effectors predicted by SecReT6 in all *E. bugandensis* and *E. cloacae* strains included in this study[a].

Strain	Locus tag	Coordinates (Strand)	Best hit effector[b] (Function type)[c]	Identities (%)	E-value	Bit score	Ha-value
E. bugandensis E104107	OQ482_RS00915	187340..187552 (+)	EFF01477 (Tse)	76.1	1.07E-36	112	0.771
	OQ482_RS02615	541217..541579 (-)	EFF00045 (Tae)	43.1	3.08E-25	87.4	0.442
	OQ482_RS02695	553503..553988 (+)	EFF01309 (-)	57.5	1.12E-68	200	0.571
	OQ482_RS05430	1142084..1143469 (+)	EFF01485 (Tde)	53	2.49E-170	482	0.536
	OQ482_RS05645	1187720..1188211 (-)	EFF01309 (-)	70.6	6.94E-89	251	0.706
	OQ482_RS06005	1263097..1263306 (+)	EFF01478 (Tse)	72.1	3.48E-34	106	0.71
	OQ482_RS07350	1537600..1539252 (-)	EFF01841 (-)	92.9	0	1069	0.929
	OQ482_RS07385	1547359..1547580 (-)	EFF01478 (Tse)	53.1	2.71E-22	76.3	0.466
	OQ482_RS07400	1550579..1551058 (-)	EFF01309 (-)	54.6	4.00E-64	189	0.56
	OQ482_RS10510	2178032..2180455 (-)	EFF01467 (Tde)	60	0	931	0.559
	OQ482_RS10680	2234528..2235400 (-)	EFF01453 (Tse)	81.9	0	495	0.81
	OQ482_RS11090	2311726..2312211 (-)	EFF01309 (-)	61	7.62E-74	213	0.621
	OQ482_RS11590	2424566..2424928 (+)	EFF00045 (Tae)	44.6	2.08E-30	100	0.45
	OQ482_RS11805	2467686..2468168 (+)	EFF01309 (-)	71.2	6.93E-87	246	0.713
	OQ482_RS11885	2481170..2481382 (-)	EFF01478 (Tse)	53.7	1.17E-20	72	0.514
	OQ482_RS11890	2481670..2481888 (+)	EFF01478 (Tse)	59.2	1.00E-26	87.4	0.583
	OQ482_RS12840	2666708..2671132 (-)	EFF01254 (Tse)	94.9	0	2539	0.855
	OQ482_RS12865	2673887..2676472 (-)	EFF01840 (-)	76.2	0	1346	0.763
	OQ482_RS12890	2679092..2680354 (-)	EFF01495 (Tre)	66.5	4.91E-79	246	0.436
	OQ482_RS12900	2681163..2683091 (-)	EFF01840 (-)	77.4	0	1041	0.774
	OQ482_RS12960	2696241..2696732 (-)	EFF01418 (Tae)	96.3	7.26E-121	332	0.963
	OQ482_RS12965	2696791..2697273 (-)	EFF00505 (-)	100	2.10E-118	326	1
	OQ482_RS13245	2759410..2759619 (-)	EFF01478 (Tse)	73.1	1.34E-33	104	0.71
	OQ482_RS13720	2856431..2856922 (+)	EFF01309 (-)	69.9	1.90E-87	248	0.699
	OQ482_RS14650	3061128..3062093 (+)	EFF01504 (Tse)	43.6	9.97E-86	256	0.433
	OQ482_RS15995	3339969..3340460 (-)	EFF00560 (-)	93.9	9.97E-109	301	0.939
	OQ482_RS19625	4096501..4096986 (+)	EFF01309 (-)	60.4	8.92E-72	208	0.615
E. bugandensis E105227	OQ483_RS00400	76532..77017 (-)	EFF01309 (-)	56.9	7.15E-66	193	0.565
	OQ483_RS00950	193448..193660 (+)	EFF01477 (Tse)	76.1	1.07E-36	112	0.771
	OQ483_RS03370	689496..689975 (-)	EFF00429 (-)	50.9	5.36E-58	173	0.516
	OQ483_RS06170	1290879..1292264 (+)	EFF01485 (Tde)	53	2.49E-170	482	0.536
	OQ483_RS06295	1316161..1316640 (-)	EFF01309 (-)	49.7	1.34E-59	177	0.509
	OQ483_RS06430	1342888..1347099 (+)	EFF01255 (Tse)	95.2	0	2429	0.872

	OQ483_RS06825	1430407..1430616 (+)	EFF01478 (Tse)	72.1	3.48E-34	106	0.71
	OQ483_RS08185	1713732..1715384 (-)	EFF01841 (-)	92.9	0	1069	0.929
	OQ483_RS08220	1723485..1723706 (-)	EFF01478 (Tse)	53.1	2.71E-22	76.3	0.466
	OQ483_RS11400	2361367..2361849 (-)	EFF01309 (-)	62.6	1.13E-72	210	0.638
	OQ483_RS11790	2452770..2453642 (-)	EFF01453 (Tse)	81.9	0	495	0.81
	OQ483_RS12435	2587599..2588084 (+)	EFF01309 (-)	69.4	1.56E-87	248	0.689
	OQ483_RS12620	2631470..2631949 (+)	EFF00429 (-)	53.1	5.18E-61	181	0.535
	OQ483_RS12890	2685652..2685870 (+)	EFF01478 (Tse)	59.2	1.81E-27	89.4	0.583
	OQ483_RS13310	2775328..2775540 (-)	EFF01477 (Tse)	74.6	2.40E-36	111	0.757
	OQ483_RS13695	2853522..2857985 (-)	EFF01254 (Tse)	94	0	2519	0.84
	OQ483_RS13710	2859210..2861771 (-)	EFF01840 (-)	85.6	0	1158	0.641
	OQ483_RS13785	2876945..2877436 (-)	EFF01418 (Tae)	96.3	7.26E-121	332	0.963
	OQ483_RS13790	2877495..2877977 (-)	EFF00505 (-)	100	2.10E-118	326	1
	OQ483_RS14075	2941524..2941733 (-)	EFF01478 (Tse)	73.1	1.34E-33	104	0.71
	OQ483_RS14555	3039831..3040190 (+)	EFF00045 (Tae)	44.7	1.86E-26	90.5	0.462
	OQ483_RS15625	3261121..3262086 (+)	EFF01504 (Tse)	43.3	1.14E-85	256	0.43
	OQ483_RS16690	3501086..3503716 (-)	EFF01826 (Tse)	60.8	0	959	0.531
	OQ483_RS16705	3507464..3507955 (-)	EFF00560 (-)	94.5	3.40E-109	303	0.945
E. bugandensis 220	FBF69_RS00815	170933..171145 (-)	EFF01477 (Tse)	76.1	1.07E-36	112	0.771
	FBF69_RS03670	775412..777979 (+)	EFF01826 (Tse)	46.7	0	679	0.442
	FBF69_RS04945	1006726..1007211 (-)	EFF01309 (-)	60.4	8.92E-72	208	0.615
	FBF69_RS08745	1770890..1771381 (+)	EFF00560 (-)	94.5	3.40E-109	303	0.945
	FBF69_RS08755	1774192..1776624 (+)	EFF01826 (Tse)	45.7	0	660	0.448
	FBF69_RS10665	2199424..2199783 (-)	EFF00045 (Tae)	44.7	1.01E-26	90.9	0.462
	FBF69_RS11140	2296604..2296813 (+)	EFF01478 (Tse)	73.1	1.34E-33	104	0.71
	FBF69_RS11420	2358930..2359412 (+)	EFF00505 (-)	100	2.10E-118	326	1
	FBF69_RS11425	2359471..2359962 (+)	EFF01418 (Tae)	96.3	7.26E-121	332	0.963
	FBF69_RS11485	2373109..2375067 (+)	EFF01839 (-)	80.6	0	951	0.687
	FBF69_RS11525	2381247..2383787 (+)	EFF01840 (-)	71.8	0	1254	0.73
	FBF69_RS11550	2386508..2390887 (+)	EFF01254 (Tse)	92.1	0	2531	0.866
	FBF69_RS24160	2403762..2403884 (+)	EFF01255 (Tse)	52.9	1.51E-11	50.4	0.45
	FBF69_RS11710	2408166..2408657 (+)	EFF01516 (Tse)	46.9	2.19E-46	144	0.466
	FBF69_RS12800	2619389..2619607 (-)	EFF01478 (Tse)	59.2	1.81E-27	89.4	0.583
	FBF69_RS12805	2619895..2620107 (+)	EFF01478 (Tse)	53.7	1.09E-20	72	0.514
	FBF69_RS12880	2633104..2633586 (-)	EFF01309 (-)	71.2	6.93E-87	246	0.713
	FBF69_RS13095	2676343..2676705 (-)	EFF00045 (Tae)	43.8	7.35E-30	99	0.442
	FBF69_RS13220	2705591..2705959 (+)	EFF00045 (Tae)	52.1	8.67E-37	116	0.516
	FBF69_RS13285	2720207..2720686 (-)	EFF00429 (-)	50.9	9.50E-60	177	0.516

	FBF69_RS14015	2870841..2871713 (+)	EFF01453 (Tse)	81.9	0	495	0.81
	FBF69_RS14185	2925782..2928205 (+)	EFF01467 (Tde)	60.8	0	940	0.566
	FBF69_RS17345	3553262..3553483 (+)	EFF01478 (Tse)	53.1	2.71E-22	76.3	0.466
	FBF69_RS17380	3561590..3563242 (+)	EFF01841 (-)	92.5	0	1066	0.925
	FBF69_RS18695	3827792..3828001 (-)	EFF01478 (Tse)	72.1	3.48E-34	106	0.71
	FBF69_RS19085	3909288..3909779 (+)	EFF01309 (-)	69.9	7.82E-88	249	0.699
	FBF69_RS19300	3953956..3955341 (-)	EFF01485 (Tde)	53	2.49E-170	482	0.536
	FBF69_RS21580	4452070..4452561 (-)	EFF00429 (-)	45	1.20E-55	167	0.442
	FBF69_RS21995	4534735..4535226 (-)	EFF01309 (-)	55.2	4.02E-61	181	0.552
	FBF69_RS22265	4586784..4587146 (+)	EFF00045 (Tae)	43.1	1.95E-24	85.1	0.442
E. bugandensis 1367	FBF68_RS00530	100563..100772 (-)	EFF01478 (Tse)	72.1	3.48E-34	106	0.71
	FBF68_RS00945	184713..185204 (+)	EFF01309 (-)	69.9	6.21E-88	249	0.699
	FBF68_RS01155	229403..230788 (-)	EFF01485 (Tde)	53	2.49E-170	482	0.536
	FBF68_RS05695	1184480..1184692 (-)	EFF01477 (Tse)	76.1	1.07E-36	112	0.771
	FBF68_RS13200	2729266..2729757 (+)	EFF00560 (-)	94.5	3.40E-109	303	0.945
	FBF68_RS13225	2736707..2739058 (+)	EFF01826 (Tse)	46.8	0	664	0.458
	FBF68_RS13245	2742291..2744810 (+)	EFF01826 (Tse)	44.7	0	668	0.445
	FBF68_RS15225	3178320..3178679 (-)	EFF00045 (Tae)	44.7	1.01E-26	90.9	0.462
	FBF68_RS15830	3291257..3291466 (+)	EFF01478 (Tse)	73.1	1.34E-33	104	0.71
	FBF68_RS16115	3354879..3355361 (+)	EFF00505 (-)	100	2.10E-118	326	1
	FBF68_RS16120	3355420..3355911 (+)	EFF01418 (Tae)	96.3	7.26E-121	332	0.963
	FBF68_RS16180	3369058..3371016 (+)	EFF01839 (-)	80.6	0	951	0.687
	FBF68_RS16220	3377196..3379736 (+)	EFF01840 (-)	72	0	1258	0.733
	FBF68_RS16245	3382457..3386881 (+)	EFF01254 (Tse)	94.4	0	2529	0.85
	FBF68_RS17580	3654684..3655046 (-)	EFF00045 (Tae)	44.6	2.08E-30	100	0.45
	FBF68_RS17775	3698682..3699167 (-)	EFF01309 (-)	70	3.54E-88	249	0.696
	FBF68_RS18500	3844029..3844901 (+)	EFF01453 (Tse)	81.9	0	495	0.81
	FBF68_RS22095	4574491..4574712 (+)	EFF01478 (Tse)	53.1	2.71E-22	76.3	0.466
	FBF68_RS22130	4582818..4584470 (+)	EFF01841 (-)	92.9	0	1069	0.929
E. bugandensis STN0717-56	JLD64_RS00160	32764..33243 (+)	EFF00429 (-)	50.9	8.38E-61	180	0.516
	JLD64_RS00895	176710..176922 (+)	EFF01477 (Tse)	76.1	1.07E-36	112	0.771
	JLD64_RS02670	555547..556032 (+)	EFF01309 (-)	57.5	1.12E-68	200	0.571
	JLD64_RS02890	599606..600097 (+)	EFF01309 (-)	55.2	2.43E-61	182	0.552
	JLD64_RS05430	1152107..1153492 (+)	EFF01485 (Tde)	53	2.49E-170	482	0.536
	JLD64_RS05650	1198973..1199464 (-)	EFF01309 (-)	69.9	6.21E-88	249	0.699
	JLD64_RS06065	1285922..1286131 (+)	EFF01478 (Tse)	72.1	3.48E-34	106	0.71
	JLD64_RS07380	1553091..1554743 (-)	EFF01841 (-)	92.5	0	1065	0.925
	JLD64_RS07420	1564136..1564357 (-)	EFF01478 (Tse)	53.1	2.71E-22	76.3	0.466

	JLD64_RS10665	2241344..2242216 (-)	EFF01453 (Tse)	81.9	0	495	0.81
	JLD64_RS11060	2316417..2316902 (-)	EFF01309 (-)	61	1.01E-73	213	0.621
	JLD64_RS11550	2427400..2427762 (+)	EFF00045 (Tae)	44.6	2.08E-30	100	0.45
	JLD64_RS11850	2483997..2484209 (-)	EFF01478 (Tse)	52.2	9.74E-19	67.4	0.5
	JLD64_RS11855	2484497..2484715 (+)	EFF01478 (Tse)	59.2	1.81E-27	89.4	0.583
	JLD64_RS12795	2679983..2684416 (-)	EFF01254 (Tse)	94.5	0	2531	0.852
	JLD64_RS12820	2687137..2689677 (-)	EFF01840 (-)	71.9	0	1257	0.732
	JLD64_RS12860	2695857..2697815 (-)	EFF01839 (-)	80.9	0	952	0.69
	JLD64_RS12920	2710965..2711456 (-)	EFF01418 (Tae)	96.3	7.26E-121	332	0.963
	JLD64_RS12925	2711515..2711997 (-)	EFF00505 (-)	100	2.10E-118	326	1
	JLD64_RS13205	2774132..2774341 (-)	EFF01478 (Tse)	73.1	1.34E-33	104	0.71
	JLD64_RS13680	2871159..2871518 (+)	EFF00045 (Tae)	45.5	2.95E-27	92.4	0.471
	JLD64_RS15685	3314928..3317558 (-)	EFF01826 (Tse)	60.8	0	961	0.531
	JLD64_RS15700	3321310..3321801 (-)	EFF00560 (-)	94.5	3.40E-109	303	0.945
E. bugandensis FDAARGOS 1427	I6L57_RS00680	137945..138157 (+)	EFF01477 (Tse)	76.1	1.07E-36	112	0.771
	I6L57_RS02345	488449..488811 (-)	EFF00045 (Tae)	43.1	4.96E-25	86.7	0.442
	I6L57_RS02565	539316..539801 (+)	EFF01309 (-)	57.5	1.02E-68	200	0.571
	I6L57_RS03200	660312..660803 (+)	EFF00429 (-)	45	1.20E-55	167	0.442
	I6L57_RS05345	1133405..1134790 (+)	EFF01485 (Tde)	53	2.49E-170	482	0.536
	I6L57_RS05560	1178986..1179477 (-)	EFF01309 (-)	69.9	5.33E-88	249	0.699
	I6L57_RS05960	1260707..1260916 (+)	EFF01478 (Tse)	72.1	3.48E-34	106	0.71
	I6L57_RS07235	1522564..1524216 (-)	EFF01841 (-)	92.7	0	1068	0.927
	I6L57_RS07270	1532322..1532543 (-)	EFF01478 (Tse)	53.1	2.71E-22	76.3	0.466
	I6L57_RS07285	1535541..1536020 (-)	EFF00429 (-)	53.4	3.22E-62	184	0.541
	I6L57_RS10530	2208107..2208979 (-)	EFF01453 (Tse)	82.2	0	495	0.814
	I6L57_RS12075	2523883..2526426 (-)	EFF01467 (Tde)	76.7	0	1189	0.669
	I6L57_RS12140	2542492..2544888 (-)	EFF01467 (Tde)	51.9	0	778	0.49
	I6L57_RS12150	2547676..2548167 (-)	EFF00560 (-)	94.5	6.56E-110	305	0.945
	I6L57_RS12940	2708287..2712720 (-)	EFF01254 (Tse)	93.9	0	2518	0.846
	I6L57_RS12965	2715441..2717981 (-)	EFF01840 (-)	71.9	0	1258	0.732
	I6L57_RS12995	2722564..2724522 (-)	EFF01839 (-)	80.2	0	946	0.684
	I6L57_RS13055	2737672..2738163 (-)	EFF01418 (Tae)	96.3	7.26E-121	332	0.963
	I6L57_RS13060	2738222..2738704 (-)	EFF00505 (-)	100	2.10E-118	326	1
	I6L57_RS13340	2800847..2801056 (-)	EFF01478 (Tse)	73.1	1.34E-33	104	0.71
	I6L57_RS13815	2897802..2898161 (+)	EFF00045 (Tae)	43.9	2.78E-25	87.4	0.454
	I6L57_RS15755	3331336..3331827 (-)	EFF00560 (-)	94.5	3.40E-109	303	0.945
	I6L57_RS17975	3808104..3808595 (+)	EFF01309 (-)	69.9	1.84E-86	245	0.699
E. bugandensis XL95	K9O83_RS00925	190166..190378 (+)	EFF01477 (Tse)	76.1	1.07E-36	112	0.771

	K9O83_RS01720	363520..366252 (+)	EFF01467 (Tde)	87	0	1396	0.736
	K9O83_RS02610	547290..547652 (-)	EFF00045 (Tae)	43.1	3.62E-25	87	0.442
	K9O83_RS05490	1168594..1169979 (+)	EFF01485 (Tde)	53	1.34E-170	483	0.536
	K9O83_RS05705	1214793..1215284 (-)	EFF01309 (-)	70.6	7.66E-89	251	0.706
	K9O83_RS06120	1298695..1298904 (+)	EFF01478 (Tse)	72.1	3.48E-34	106	0.71
	K9O83_RS07400	1560611..1562263 (-)	EFF01841 (-)	92.9	0	1069	0.929
	K9O83_RS07435	1570369..1570590 (-)	EFF01478 (Tse)	53.1	2.71E-22	76.3	0.466
	K9O83_RS09525	1988771..1990459 (-)	EFF01465 (Tle)	56.2	0	624	0.557
	K9O83_RS10710	2251081..2251953 (-)	EFF01453 (Tse)	81.9	0	495	0.81
	K9O83_RS11120	2328078..2328563 (-)	EFF01309 (-)	61	1.01E-73	213	0.621
	K9O83_RS11880	2491478..2491690 (-)	EFF01478 (Tse)	53.7	1.09E-20	72	0.514
	K9O83_RS11885	2491978..2492196 (+)	EFF01478 (Tse)	59.2	1.00E-26	87.4	0.583
	K9O83_RS12270	2556826..2559369 (-)	EFF01467 (Tde)	77	0	1192	0.672
	K9O83_RS12335	2575425..2577821 (-)	EFF01467 (Tde)	51.9	0	778	0.49
	K9O83_RS12345	2580609..2581100 (-)	EFF00560 (-)	94.5	6.56E-110	305	0.945
	K9O83_RS13110	2741714..2746114 (-)	EFF01254 (Tse)	93.9	0	2530	0.855
	K9O83_RS13135	2748835..2751375 (-)	EFF01840 (-)	72.1	0	1260	0.734
	K9O83_RS13175	2757556..2759514 (-)	EFF01839 (-)	80.6	0	949	0.687
	K9O83_RS13235	2772664..2773155 (-)	EFF01418 (Tae)	96.3	7.26E-121	332	0.963
	K9O83_RS13240	2773214..2773696 (-)	EFF00505 (-)	100	2.10E-118	326	1
	K9O83_RS13520	2835817..2836026 (-)	EFF01478 (Tse)	73.1	1.34E-33	104	0.71
	K9O83_RS14000	2934126..2934617 (+)	EFF01309 (-)	69.9	3.59E-87	247	0.699
	K9O83_RS16055	3383301..3385775 (-)	EFF01826 (Tse)	46.2	0	656	0.433
	K9O83_RS16080	3391052..3393493 (-)	EFF01826 (Tse)	46.8	0	662	0.443
	K9O83_RS16090	3396304..3396795 (-)	EFF00560 (-)	94.5	3.40E-109	303	0.945
	K9O83_RS16610	3506780..3507271 (+)	EFF01309 (-)	61.4	6.97E-73	211	0.614
	K9O83_RS20580	4315313..4317880 (-)	EFF01826 (Tse)	46.6	0	677	0.441
E. bugandensis FDAARGOS_1520	LA316_RS04160	857172..857663 (-)	EFF01309 (-)	69.9	1.84E-86	245	0.699
	LA316_RS06395	1333940..1334431 (+)	EFF00560 (-)	94.5	3.40E-109	303	0.945
	LA316_RS08340	1767606..1767965 (-)	EFF00045 (Tae)	43.9	2.78E-25	87.4	0.454
	LA316_RS08815	1864711..1864920 (+)	EFF01478 (Tse)	73.1	1.34E-33	104	0.71
	LA316_RS09095	1927063..1927545 (+)	EFF00505 (-)	100	2.10E-118	326	1
	LA316_RS09100	1927604..1928095 (+)	EFF01418 (Tae)	96.3	7.26E-121	332	0.963
	LA316_RS09160	1941245..1943203 (+)	EFF01839 (-)	80.2	0	946	0.684
	LA316_RS09195	1947786..1950326 (+)	EFF01840 (-)	71.9	0	1258	0.732
	LA316_RS09220	1953047..1957480 (+)	EFF01254 (Tse)	93.9	0	2518	0.846
	LA316_RS10025	2117600..2118091 (+)	EFF00560 (-)	94.5	6.56E-110	305	0.945
	LA316_RS10035	2120879..2123275 (+)	EFF01467 (Tde)	51.9	0	778	0.49

	LA316_RS10100	2139341..2141884 (+)	EFF01467 (Tde)	76.7	0	1189	0.669
	LA316_RS11680	2456788..2457660 (+)	EFF01453 (Tse)	82.2	0	495	0.814
	LA316_RS14935	3129747..3130226 (+)	EFF00429 (-)	53.4	3.22E-62	184	0.541
	LA316_RS14950	3133224..3133445 (+)	EFF01478 (Tse)	53.1	2.71E-22	76.3	0.466
	LA316_RS14985	3141551..3143203 (+)	EFF01841 (-)	92.7	0	1068	0.927
	LA316_RS16260	3404851..3405060 (-)	EFF01478 (Tse)	72.1	3.48E-34	106	0.71
	LA316_RS16660	3486290..3486781 (+)	EFF01309 (-)	69.9	5.33E-88	249	0.699
	LA316_RS16875	3530977..3532362 (-)	EFF01485 (Tde)	53	2.49E-170	482	0.536
	LA316_RS19025	4004964..4005455 (-)	EFF00429 (-)	45	1.20E-55	167	0.442
	LA316_RS19660	4125966..4126451 (-)	EFF01309 (-)	57.5	1.02E-68	200	0.571
	LA316_RS19880	4176956..4177318 (+)	EFF00045 (Tae)	43.1	4.96E-25	86.7	0.442
	LA316_RS21545	4527610..4527822 (-)	EFF01477 (Tse)	76.1	1.07E-36	112	0.771
E. bugandensis FDAARGOS_1497	LA318_RS01655	347128..347490 (-)	EFF00045 (Tae)	43.1	4.96E-25	86.7	0.442
	LA318_RS01875	397995..398480 (+)	EFF01309 (-)	57.5	1.02E-68	200	0.571
	LA318_RS02510	518991..519482 (+)	EFF00429 (-)	45	1.20E-55	167	0.442
	LA318_RS04660	992084..993469 (+)	EFF01485 (Tde)	53	2.49E-170	482	0.536
	LA318_RS04875	1037665..1038156 (-)	EFF01309 (-)	69.9	5.33E-88	249	0.699
	LA318_RS05275	1119386..1119595 (+)	EFF01478 (Tse)	72.1	3.48E-34	106	0.71
	LA318_RS06550	1381243..1382895 (-)	EFF01841 (-)	92.7	0	1068	0.927
	LA318_RS06585	1391001..1391222 (-)	EFF01478 (Tse)	53.1	2.71E-22	76.3	0.466
	LA318_RS06600	1394220..1394699 (-)	EFF00429 (-)	53.4	3.22E-62	184	0.541
	LA318_RS09850	2066786..2067658 (-)	EFF01453 (Tse)	82.2	0	495	0.814
	LA318_RS11420	2382562..2385105 (-)	EFF01467 (Tde)	76.7	0	1189	0.669
	LA318_RS11485	2401171..2403567 (-)	EFF01467 (Tde)	51.9	0	778	0.49
	LA318_RS11495	2406355..2406846 (-)	EFF00560 (-)	94.5	6.56E-110	305	0.945
	LA318_RS12300	2566966..2571399 (-)	EFF01254 (Tse)	93.9	0	2518	0.846
	LA318_RS12325	2574120..2576660 (-)	EFF01840 (-)	71.9	0	1258	0.732
	LA318_RS12355	2581243..2583201 (-)	EFF01839 (-)	80.2	0	946	0.684
	LA318_RS12415	2596351..2596842 (-)	EFF01418 (Tae)	96.3	7.26E-121	332	0.963
	LA318_RS12420	2596901..2597383 (-)	EFF00505 (-)	100	2.10E-118	326	1
	LA318_RS12700	2659526..2659735 (-)	EFF01478 (Tse)	73.1	1.34E-33	104	0.71
	LA318_RS13175	2756481..2756840 (+)	EFF00045 (Tae)	43.9	2.78E-25	87.4	0.454
	LA318_RS15120	3190015..3190506 (-)	EFF00560 (-)	94.5	3.40E-109	303	0.945
	LA318_RS17350	3666783..3667274 (+)	EFF01309 (-)	69.9	1.84E-86	245	0.699
	LA318_RS22450	4720509..4720721 (+)	EFF01477 (Tse)	76.1	1.07E-36	112	0.771
E. bugandensis FDAARGOS_1496	LA326_RS03340	680862..681353 (-)	EFF01309 (-)	69.9	1.84E-86	245	0.699
	LA326_RS05570	1157630..1158121 (+)	EFF00560 (-)	94.5	3.40E-109	303	0.945
	LA326_RS07515	1591296..1591655 (-)	EFF00045 (Tae)	43.9	2.78E-25	87.4	0.454

	LA326_RS07990	1688401..1688610 (+)	EFF01478 (Tse)	73.1	1.34E-33	104	0.71
	LA326_RS08270	1750753..1751235 (+)	EFF00505 (-)	100	2.10E-118	326	1
	LA326_RS08275	1751294..1751785 (+)	EFF01418 (Tae)	96.3	7.26E-121	332	0.963
	LA326_RS08335	1764935..1766893 (+)	EFF01839 (-)	80.2	0	946	0.684
	LA326_RS08365	1771476..1774016 (+)	EFF01840 (-)	71.9	0	1258	0.732
	LA326_RS08390	1776737..1781170 (+)	EFF01254 (Tse)	93.9	0	2518	0.846
	LA326_RS09195	1941290..1941781 (+)	EFF00560 (-)	94.5	6.56E-110	305	0.945
	LA326_RS09205	1944569..1946965 (+)	EFF01467 (Tde)	51.9	0	778	0.49
	LA326_RS09270	1963031..1965574 (+)	EFF01467 (Tde)	76.7	0	1189	0.669
	LA326_RS10845	2280478..2281350 (+)	EFF01453 (Tse)	82.2	0	495	0.814
	LA326_RS14095	2953437..2953916 (+)	EFF00429 (-)	53.4	3.22E-62	184	0.541
	LA326_RS14110	2956914..2957135 (+)	EFF01478 (Tse)	53.1	2.71E-22	76.3	0.466
	LA326_RS14145	2965241..2966893 (+)	EFF01841 (-)	92.7	0	1068	0.927
	LA326_RS15420	3228541..3228750 (-)	EFF01478 (Tse)	72.1	3.48E-34	106	0.71
	LA326_RS15820	3309980..3310471 (+)	EFF01309 (-)	69.9	5.33E-88	249	0.699
	LA326_RS16035	3354667..3356052 (-)	EFF01485 (Tde)	53	2.49E-170	482	0.536
	LA326_RS18185	3828654..3829145 (-)	EFF00429 (-)	45	1.20E-55	167	0.442
	LA326_RS18820	3949656..3950141 (-)	EFF01309 (-)	57.5	1.02E-68	200	0.571
	LA326_RS19040	4000646..4001008 (+)	EFF00045 (Tae)	43.1	4.96E-25	86.7	0.442
	LA326_RS20705	4351300..4351512 (-)	EFF01477 (Tse)	76.1	1.07E-36	112	0.771
E. bugandensis CMCC(B)45301	M1V99_RS01470	191086..191298 (+)	EFF01477 (Tse)	76.1	1.07E-36	112	0.771
	M1V99_RS05990	1148150..1149535 (+)	EFF01485 (Tde)	53	1.27E-170	483	0.536
	M1V99_RS06580	1273214..1273423 (+)	EFF01478 (Tse)	72.1	3.48E-34	106	0.71
	M1V99_RS07855	1533851..1535503 (-)	EFF01841 (-)	92.7	0	1066	0.927
	M1V99_RS07890	1543610..1543831 (-)	EFF01478 (Tse)	53.1	2.71E-22	76.3	0.466
	M1V99_RS11110	2214523..2215395 (-)	EFF01453 (Tse)	81.9	0	495	0.81
	M1V99_RS11865	2370375..2370743 (-)	EFF00045 (Tae)	52.1	8.67E-37	116	0.516
	M1V99_RS12000	2400113..2400475 (+)	EFF00045 (Tae)	44.6	2.08E-30	100	0.45
	M1V99_RS13010	2609702..2614117 (-)	EFF01254 (Tse)	94.9	0	2548	0.858
	M1V99_RS13035	2616872..2619457 (-)	EFF01840 (-)	76	0	1345	0.761
	M1V99_RS13070	2624149..2626077 (-)	EFF01840 (-)	77.6	0	1043	0.776
	M1V99_RS13130	2639227..2639718 (-)	EFF01418 (Tae)	96.3	7.26E-121	332	0.963
	M1V99_RS13135	2639777..2640259 (-)	EFF00505 (-)	100	2.10E-118	326	1
	M1V99_RS13415	2702393..2702602 (-)	EFF01478 (Tse)	73.1	1.34E-33	104	0.71
	M1V99_RS13890	2799413..2799904 (+)	EFF01309 (-)	69.3	1.56E-86	245	0.693
	M1V99_RS16165	3302922..3303413 (-)	EFF00560 (-)	94.5	3.40E-109	303	0.945
	M1V99_RS19545	4019704..4020189 (+)	EFF01309 (-)	60.4	8.92E-72	208	0.615
E. bugandensis EB-247	DG357_RS00945	193074..193286 (+)	EFF01477 (Tse)	76.1	1.07E-36	112	0.771

	DG357_RS02635	543560..543922 (-)	EFF00045 (Tae)	43.1	4.96E-25	86.7	0.442
	DG357_RS02865	594423..594908 (+)	EFF01309 (-)	57.5	1.02E-68	200	0.571
	DG357_RS03500	715419..715910 (+)	EFF00429 (-)	45	1.20E-55	167	0.442
	DG357_RS05695	1188514..1189899 (+)	EFF01485 (Tde)	53	2.49E-170	482	0.536
	DG357_RS05905	1234095..1234586 (-)	EFF01309 (-)	69.9	5.33E-88	249	0.699
	DG357_RS07640	1577660..1579312 (-)	EFF01841 (-)	92.7	0	1068	0.927
	DG357_RS07675	1587418..1587639 (-)	EFF01478 (Tse)	53.1	2.71E-22	76.3	0.466
	DG357_RS07690	1590637..1591116 (-)	EFF00429 (-)	53.4	3.22E-62	184	0.541
	DG357_RS11030	2263158..2264030 (-)	EFF01453 (Tse)	82.2	0	495	0.814
	DG357_RS12580	2578925..2581468 (-)	EFF01467 (Tde)	76.7	0	1189	0.669
	DG357_RS12635	2596551..2597042 (-)	EFF00560 (-)	94.5	6.56E-110	305	0.945
	DG357_RS13440	2757160..2761593 (-)	EFF01254 (Tse)	93.9	0	2518	0.846
	DG357_RS13465	2764314..2766854 (-)	EFF01840 (-)	71.9	0	1258	0.732
	DG357_RS13500	2771437..2773395 (-)	EFF01839 (-)	80.2	0	946	0.684
	DG357_RS13560	2786545..2787036 (-)	EFF01418 (Tae)	96.3	7.26E-121	332	0.963
	DG357_RS13565	2787095..2787577 (-)	EFF00505 (-)	100	2.10E-118	326	1
	DG357_RS13855	2849718..2849927 (-)	EFF01478 (Tse)	73.1	1.34E-33	104	0.71
	DG357_RS14350	2946672..2947031 (+)	EFF00045 (Tae)	43.9	2.78E-25	87.4	0.454
	DG357_RS16290	3380199..3380690 (-)	EFF00560 (-)	94.5	3.40E-109	303	0.945
	DG357_RS18590	3856967..3857458 (+)	EFF01309 (-)	69.9	1.84E-86	245	0.699
E. cloacae 1382	LQ108_RS01030	215409..215621 (+)	EFF01477 (Tse)	76.1	1.07E-36	112	0.771
	LQ108_RS02685	570884..571381 (+)	EFF01309 (-)	57.7	3.55E-71	207	0.57
	LQ108_RS02875	607387..607878 (+)	EFF01309 (-)	55.2	2.33E-61	182	0.552
	LQ108_RS05225	1110998..1112383 (+)	EFF01485 (Tde)	52.8	1.36E-169	480	0.534
	LQ108_RS05490	1166998..1171194 (+)	EFF01255 (Tse)	94.5	0	2499	0.914
	LQ108_RS05530	1175751..1176191 (+)	EFF01255 (Tse)	95.7	2.06E-45	152	0.459
	LQ108_RS06245	1307997..1308206 (+)	EFF01478 (Tse)	72.1	3.48E-34	106	0.71
	LQ108_RS07575	1580241..1581893 (-)	EFF01841 (-)	92.7	0	1073	0.927
	LQ108_RS07610	1590056..1590277 (-)	EFF01478 (Tse)	54.7	1.42E-22	77	0.479
	LQ108_RS09565	1991377..1991868 (-)	EFF01309 (-)	60	2.64E-71	207	0.589
	LQ108_RS10510	2192234..2192716 (-)	EFF01309 (-)	69.4	4.12E-82	234	0.694
	LQ108_RS11025	2312724..2313596 (-)	EFF01453 (Tse)	82.6	0	499	0.817
	LQ108_RS11725	2461244..2461726 (+)	EFF01309 (-)	68.8	3.51E-85	242	0.688
	LQ108_RS12315	2597410..2597901 (-)	EFF00560 (-)	94.5	2.64E-109	303	0.945
	LQ108_RS12510	2641283..2641495 (-)	EFF01478 (Tse)	52.2	2.15E-19	68.9	0.5
	LQ108_RS12515	2641779..2641997 (+)	EFF01478 (Tse)	60.6	1.23E-28	92.4	0.597
	LQ108_RS13380	2810815..2812155 (-)	EFF01495 (Tre)	62.3	3.06E-160	455	0.623
	LQ108_RS13390	2812964..2814892 (-)	EFF01840 (-)	77.6	0	1042	0.776

	LQ108_RS13460	2829201..2829743 (-)	EFF00144 (-)	43.5	1.03E-37	131	0.428
	LQ108_RS13465	2829801..2830283 (-)	EFF00505 (-)	100	2.10E-118	326	1
	LQ108_RS13555	2850860..2851339 (+)	EFF00429 (-)	54	6.14E-62	183	0.547
	LQ108_RS13770	2897851..2898060 (-)	EFF01478 (Tse)	73.1	1.34E-33	104	0.71
	LQ108_RS14255	2995256..2995747 (+)	EFF01309 (-)	66.9	3.62E-84	239	0.656
	LQ108_RS17585	3714114..3714599 (-)	EFF01309 (-)	62.2	4.05E-75	216	0.634
E. cloacae ATCC 13047	ECL_RS00165	31683..32162 (+)	EFF00429 (-)	50.3	1.18E-61	182	0.509
	ECL_RS01030	212839..213051 (+)	EFF01477 (Tse)	76.1	1.07E-36	112	0.771
	ECL_RS06205	1309470..1310855 (+)	EFF01485 (Tde)	52.6	6.63E-169	478	0.531
	ECL_RS06785	1397170..1397652 (-)	EFF01309 (-)	69.4	4.05E-85	242	0.694
	ECL_RS07270	1497639..1497848 (+)	EFF01478 (Tse)	73.1	1.34E-33	104	0.71
	ECL_RS07555	1562931..1563413 (+)	EFF00505 (-)	100	2.10E-118	326	1
	ECL_RS07560	1563472..1563963 (+)	EFF01418 (Tae)	100	4.26E-124	340	1
	ECL_RS07615	1576408..1577826 (+)	EFF01872 (Tle)	100	0	972	1
	ECL_RS07645	1582007..1583935 (+)	EFF01840 (-)	77.4	0	1041	0.774
	ECL_RS07695	1591543..1595931 (+)	EFF01254 (Tse)	100	0	3011	1
	ECL_RS07740	1601517..1602014 (+)	EFF01516 (Tse)	52.9	3.06E-50	154	0.448
	ECL_RS07765	1605461..1605715 (+)	EFF01254 (Tse)	77.1	1.00E-32	113	0.643
	ECL_RS10065	2080330..2081202 (+)	EFF01453 (Tse)	82.2	0	499	0.814
	ECL_RS10590	2199781..2200263 (+)	EFF01309 (-)	69.4	4.12E-82	234	0.694
	ECL_RS13605	2823490..2823711 (+)	EFF01478 (Tse)	54.7	1.42E-22	77	0.479
	ECL_RS13645	2833105..2834757 (+)	EFF01841 (-)	92.5	0	1073	0.925
	ECL_RS14390	2984348..2984662 (-)	EFF01873 (Tde)	48	2.54E-29	103	0.471
	ECL_RS15130	3128674..3128883 (-)	EFF01478 (Tse)	72.1	3.48E-34	106	0.71
	ECL_RS15470	3200343..3204599 (-)	EFF01255 (Tse)	100	0	2899	1
	ECL_RS18965	3901554..3902045 (+)	EFF01309 (-)	61.4	6.38E-73	211	0.614
	ECL_RS19875	4091478..4091963 (-)	EFF01309 (-)	61.6	3.78E-74	214	0.627
E. cloacae GTT036	EC036_RS00175	34347..34826 (+)	EFF00429 (-)	50.3	9.94E-62	182	0.509
	EC036_RS01040	218789..219001 (+)	EFF01477 (Tse)	76.1	1.07E-36	112	0.771
	EC036_RS02270	473139..473624 (+)	EFF01309 (-)	70.6	4.49E-84	239	0.702
	EC036_RS05530	1176062..1177447 (+)	EFF01485 (Tde)	52.8	1.62E-169	480	0.534
	EC036_RS05790	1232070..1236254 (+)	EFF01255 (Tse)	99	0	2499	0.905
	EC036_RS06095	1305515..1305724 (+)	EFF01478 (Tse)	72.1	3.48E-34	106	0.71
	EC036_RS07360	1564541..1566193 (-)	EFF01841 (-)	92.7	0	1073	0.927
	EC036_RS07395	1574356..1574577 (-)	EFF01478 (Tse)	54.7	1.42E-22	77	0.479
	EC036_RS10050	2135341..2135823 (-)	EFF01309 (-)	70	9.53E-87	246	0.7
	EC036_RS10670	2285000..2285872 (-)	EFF01453 (Tse)	82.2	0	497	0.814
	EC036_RS13225	2801380..2802642 (-)	EFF01495 (Tre)	64.3	7.08E-80	248	0.445

	EC036_RS13235	2803451..2805379 (-)	EFF01840 (-)	77.4	0	1042	0.774
	EC036_RS13305	2819644..2820135 (-)	EFF01418 (Tae)	100	4.26E-124	340	1
	EC036_RS13310	2820194..2820676 (-)	EFF00505 (-)	100	2.10E-118	326	1
	EC036_RS13600	2885758..2885967 (-)	EFF01478 (Tse)	73.1	1.34E-33	104	0.71
	EC036_RS14070	2982238..2982720 (+)	EFF01309 (-)	69.4	4.05E-85	242	0.694
E. cloacae M12X01451	GRI03_RS01910	210063..210275 (+)	EFF01477 (Tse)	76.1	1.07E-36	112	0.771
	GRI03_RS06460	1191278..1192663 (+)	EFF01485 (Tde)	52.8	2.43E-169	479	0.534
	GRI03_RS06710	1246509..1250720 (+)	EFF01255 (Tse)	95.8	0	2477	0.901
	GRI03_RS07050	1322883..1323092 (+)	EFF01478 (Tse)	72.1	3.48E-34	106	0.71
	GRI03_RS07955	1500696..1504760 (-)	EFF28264 (-)	65.2	0	1623	0.601
	GRI03_RS08385	1597842..1599494 (-)	EFF01841 (-)	92.5	0	1073	0.925
	GRI03_RS08420	1607657..1607878 (-)	EFF01478 (Tse)	54.7	1.42E-22	77	0.479
	GRI03_RS11390	2222583..2223065 (-)	EFF01309 (-)	70	9.53E-87	246	0.7
	GRI03_RS11545	2258938..2259420 (-)	EFF01309 (-)	69.4	4.12E-82	234	0.694
	GRI03_RS12040	2373739..2374611 (-)	EFF01453 (Tse)	82.6	0	499	0.817
	GRI03_RS13910	2779230..2780564 (-)	EFF01495 (Tre)	60.5	1.38E-148	425	0.61
	GRI03_RS13920	2781373..2783301 (-)	EFF01840 (-)	77.4	0	1042	0.774
	GRI03_RS13945	2786215..2787681 (-)	EFF01872 (Tle)	83.6	0	835	0.834
	GRI03_RS14000	2800126..2800617 (-)	EFF01418 (Tae)	100	4.26E-124	340	1
	GRI03_RS14005	2800676..2801158 (-)	EFF00505 (-)	100	2.10E-118	326	1
	GRI03_RS14290	2866275..2866484 (-)	EFF01478 (Tse)	73.1	1.34E-33	104	0.71
	GRI03_RS14765	2962751..2963233 (+)	EFF01309 (-)	68.8	5.68E-85	241	0.688
	GRI03_RS20000	4040414..4040932 (+)	EFF01440 (-)	87.8	1.94E-116	322	0.878
	GRI03_RS20005	4041133..4042098 (+)	EFF01504 (Tse)	44.6	1.14E-97	286	0.439
	GRI03_RS22270	4490353..4494234 (+)	EFF28269 (-)	62.6	0	1537	0.607
	GRI03_RS23120	4685589..4689824 (-)	EFF28269 (-)	65.4	0	1614	0.583
	GRI03_RS24135	4907373..4911374 (-)	EFF28264 (-)	65.2	0	1615	0.611
E. cloacae PIMB10EC27	B2J95_RS00165	31683..32162 (+)	EFF00429 (-)	49.7	2.44E-61	181	0.503
	B2J95_RS01045	210967..211179 (+)	EFF01477 (Tse)	76.1	1.07E-36	112	0.771
	B2J95_RS02690	540361..540846 (+)	EFF01309 (-)	70.6	4.49E-84	239	0.702
	B2J95_RS06365	1300935..1302320 (+)	EFF01485 (Tde)	52.8	1.04E-168	478	0.534
	B2J95_RS06660	1362090..1366217 (+)	EFF01255 (Tse)	95.4	0	2504	0.925
	B2J95_RS27275	1371304..1371378 (+)	EFF01255 (Tse)	86.4	5.99E-10	45.1	0.792
	B2J95_RS07040	1442972..1443181 (+)	EFF01478 (Tse)	72.1	3.48E-34	106	0.71
	B2J95_RS08350	1705936..1707588 (-)	EFF01841 (-)	92.7	0	1073	0.927
	B2J95_RS08385	1715751..1715972 (-)	EFF01478 (Tse)	54.7	1.42E-22	77	0.479
	B2J95_RS27500	2185720..2186331 (+)	EFF01309 (-)	82.4	1.89E-65	194	0.438
	B2J95_RS11565	2346657..2347139 (-)	EFF01309 (-)	70	9.53E-87	246	0.7

	B2J95_RS12035	2459671..2460543 (-)	EFF01453 (Tse)	82.6	0	498	0.817
	B2J95_RS13300	2736618..2739059 (-)	EFF01826 (Tse)	46.7	0	668	0.439
	B2J95_RS13310	2741865..2742356 (-)	EFF00560 (-)	94.5	2.64E-109	303	0.945
	B2J95_RS14120	2911554..2915930 (-)	EFF01254 (Tse)	98.3	0	2688	0.897
	B2J95_RS14145	2918579..2920501 (-)	EFF01840 (-)	84.7	0	1144	0.848
	B2J95_RS14155	2921091..2923019 (-)	EFF01840 (-)	77.6	0	1039	0.777
	B2J95_RS14185	2927010..2928476 (-)	EFF01872 (Tle)	83.2	0	831	0.83
	B2J95_RS14240	2940921..2941412 (-)	EFF01418 (Tae)	100	4.26E-124	340	1
	B2J95_RS14245	2941471..2941953 (-)	EFF00505 (-)	100	2.10E-118	326	1
	B2J95_RS14540	3007027..3007236 (-)	EFF01478 (Tse)	73.1	1.34E-33	104	0.71
	B2J95_RS27385	3825275..3826015 (+)	EFF01521 (Tle)	50.8	2.80E-78	243	0.488
	B2J95_RS27390	3826129..3827265 (+)	EFF01521 (Tle)	42.5	4.41E-109	328	0.426
	B2J95_RS23690	4789529..4792096 (-)	EFF01826 (Tse)	47	0	682	0.446
E. cloacae CBG15936	GJ694_RS00165	32691..33170 (+)	EFF00429 (-)	50.3	1.18E-61	182	0.509
	GJ694_RS01030	216716..216928 (+)	EFF01477 (Tse)	76.1	1.07E-36	112	0.771
	GJ694_RS02240	468372..468857 (+)	EFF01309 (-)	70	4.69E-83	236	0.696
	GJ694_RS05435	1189789..1191174 (+)	EFF01485 (Tde)	52.8	4.19E-169	479	0.534
	GJ694_RS05705	1247095..1251333 (+)	EFF01255 (Tse)	99.5	0	2594	0.897
	GJ694_RS06070	1327669..1327878 (+)	EFF01478 (Tse)	72.1	3.48E-34	106	0.71
	GJ694_RS07315	1584235..1585887 (-)	EFF01841 (-)	92.7	0	1073	0.927
	GJ694_RS07350	1594050..1594271 (-)	EFF01478 (Tse)	54.7	1.42E-22	77	0.479
	GJ694_RS11230	2361384..2361866 (-)	EFF01309 (-)	68.8	4.21E-81	231	0.688
	GJ694_RS11760	2478771..2479643 (-)	EFF01453 (Tse)	82.2	0	497	0.814
	GJ694_RS12500	2622584..2623066 (+)	EFF01309 (-)	70	1.86E-85	243	0.7
	GJ694_RS13050	2752807..2753298 (-)	EFF00560 (-)	94.5	2.64E-109	303	0.945
	GJ694_RS13805	2915895..2920271 (-)	EFF01254 (Tse)	98.6	0	2701	0.9
	GJ694_RS13830	2922920..2924842 (-)	EFF01840 (-)	84.9	0	1145	0.85
	GJ694_RS13840	2925432..2927360 (-)	EFF01840 (-)	77.4	0	1040	0.774
	GJ694_RS13910	2941625..2942116 (-)	EFF01418 (Tae)	100	4.26E-124	340	1
	GJ694_RS13915	2942175..2942657 (-)	EFF00505 (-)	100	2.10E-118	326	1
	GJ694_RS13995	2962405..2962884 (+)	EFF00429 (-)	54	6.14E-62	183	0.547
	GJ694_RS14210	3009397..3009606 (-)	EFF01478 (Tse)	73.1	1.34E-33	104	0.71
	GJ694_RS14695	3106681..3107172 (+)	EFF01309 (-)	66.9	3.62E-84	239	0.656
E. cloacae DSM 30054	HWQ16_RS00015	2040..3692 (+)	EFF01841 (-)	92.5	0	1073	0.925
	HWQ16_RS00760	153285..153599 (-)	EFF01873 (Tde)	48	2.54E-29	103	0.471
	HWQ16_RS01500	297608..297817 (-)	EFF01478 (Tse)	72.1	3.48E-34	106	0.71
	HWQ16_RS01840	369277..373533 (-)	EFF01255 (Tse)	100	0	2899	1
	HWQ16_RS02450	472939..474324 (-)	EFF01485 (Tde)	52.6	6.63E-169	478	0.531

	HWQ16_RS07610	1572242..1572454 (-)	EFF01477 (Tse)	76.1	1.07E-36	112	0.771
	HWQ16_RS08480	1753132..1753611 (-)	EFF00429 (-)	50.3	1.18E-61	182	0.509
	HWQ16_RS14570	3008132..3008617 (+)	EFF01309 (-)	61.6	3.78E-74	214	0.627
	HWQ16_RS15470	3198050..3198541 (-)	EFF01309 (-)	61.4	6.38E-73	211	0.614
	HWQ16_RS18970	3882625..3883107 (-)	EFF01309 (-)	69.4	4.05E-85	242	0.694
	HWQ16_RS19455	3983095..3983304 (+)	EFF01478 (Tse)	73.1	1.34E-33	104	0.71
	HWQ16_RS19740	4048386..4048868 (+)	EFF00505 (-)	100	2.10E-118	326	1
	HWQ16_RS19745	4048927..4049418 (+)	EFF01418 (Tae)	100	4.26E-124	340	1
	HWQ16_RS19800	4061863..4063281 (+)	EFF01872 (Tle)	100	0	972	1
	HWQ16_RS19830	4067462..4069390 (+)	EFF01840 (-)	77.4	0	1041	0.774
	HWQ16_RS19855	4072428..4074350 (+)	EFF01840 (-)	84.9	0	1146	0.85
	HWQ16_RS19880	4076999..4081387 (+)	EFF01254 (Tse)	100	0	3011	1
	HWQ16_RS19925	4086973..4087470 (+)	EFF01516 (Tse)	52.9	3.06E-50	154	0.448
	HWQ16_RS19950	4090917..4091171 (+)	EFF01254 (Tse)	77.1	1.00E-32	113	0.643
	HWQ16_RS22260	4565906..4566778 (+)	EFF01453 (Tse)	82.2	0	499	0.814
	HWQ16_RS22785	4685352..4685834 (+)	EFF01309 (-)	69.4	4.12E-82	234	0.694
	HWQ16_RS25800	5309059..5309280 (+)	EFF01478 (Tse)	54.7	1.42E-22	77	0.479
E. cloacae CZ862	-	-	-	-	-	-	-
E. cloacae FDAARGOS 1431	I6L61_RS04155	887569..887781 (-)	EFF01477 (Tse)	76.1	1.07E-36	112	0.771
	I6L61_RS05025	1068460..1068939 (-)	EFF00429 (-)	50.3	1.18E-61	182	0.509
	I6L61_RS11110	2323471..2323956 (+)	EFF01309 (-)	61.6	3.78E-74	214	0.627
	I6L61_RS12010	2513390..2513881 (-)	EFF01309 (-)	61.4	6.38E-73	211	0.614
	I6L61_RS15515	3197967..3198449 (-)	EFF01309 (-)	69.4	4.05E-85	242	0.694
	I6L61_RS16000	3298437..3298646 (+)	EFF01478 (Tse)	73.1	1.34E-33	104	0.71
	I6L61_RS16285	3363728..3364210 (+)	EFF00505 (-)	100	2.10E-118	326	1
	I6L61_RS16290	3364269..3364760 (+)	EFF01418 (Tae)	100	4.26E-124	340	1
	I6L61_RS16345	3377205..3378623 (+)	EFF01872 (Tle)	100	0	972	1
	I6L61_RS16375	3382804..3384732 (+)	EFF01840 (-)	77.4	0	1041	0.774
	I6L61_RS16400	3387770..3389692 (+)	EFF01840 (-)	84.9	0	1146	0.85
	I6L61_RS16425	3392341..3396729 (+)	EFF01254 (Tse)	100	0	3011	1
	I6L61_RS16470	3402315..3402812 (+)	EFF01516 (Tse)	52.9	3.06E-50	154	0.448
	I6L61_RS16495	3406289..3406513 (+)	EFF01254 (Tse)	79.3	6.89E-26	93.2	0.622
	I6L61_RS18795	3881251..3882123 (+)	EFF01453 (Tse)	82.2	0	499	0.814
	I6L61_RS19320	4000700..4001182 (+)	EFF01309 (-)	69.4	4.12E-82	234	0.694
	I6L61_RS22340	4624414..4624635 (+)	EFF01478 (Tse)	54.7	1.42E-22	77	0.479
	I6L61_RS22380	4634030..4635682 (+)	EFF01841 (-)	92.5	0	1073	0.925
	I6L61_RS23125	4785277..4785591 (-)	EFF01873 (Tde)	48	2.54E-29	103	0.471
	I6L61_RS23860	4929605..4929814 (-)	EFF01478 (Tse)	72.1	3.48E-34	106	0.71

	I6L61_RS24200	5001275..5005531 (-)	EFF01255 (Tse)	100	0	2899	1
	I6L61_RS24810	5104938..5106323 (-)	EFF01485 (Tde)	52.6	6.63E-169	478	0.531
E. cloacae ECl_1.VN	LH408_RS00300	59205..59684 (+)	EFF00429 (-)	49.7	1.28E-60	179	0.503
	LH408_RS01200	248813..249025 (+)	EFF01477 (Tse)	76.1	1.07E-36	112	0.771
	LH408_RS02490	517086..517571 (+)	EFF01309 (-)	70.6	2.65E-83	237	0.702
	LH408_RS02895	603560..604057 (+)	EFF01309 (-)	57.7	3.55E-71	207	0.57
	LH408_RS05610	1201776..1203161 (+)	EFF01485 (Tde)	52.8	2.43E-169	479	0.534
	LH408_RS05865	1257499..1261680 (+)	EFF01255 (Tse)	98.4	0	2581	0.907
	LH408_RS06215	1333322..1333531 (+)	EFF01478 (Tse)	72.1	3.48E-34	106	0.71
	LH408_RS07510	1599141..1600793 (-)	EFF01841 (-)	92.7	0	1073	0.927
	LH408_RS07545	1608956..1609177 (-)	EFF01478 (Tse)	54.7	1.42E-22	77	0.479
	LH408_RS10475	2218125..2218607 (-)	EFF01309 (-)	69.4	4.12E-82	234	0.694
	LH408_RS10795	2276869..2277360 (-)	EFF01309 (-)	57.7	7.58E-72	208	0.577
	LH408_RS11005	2341675..2342547 (-)	EFF01453 (Tse)	82.6	0	499	0.817
	LH408_RS12205	2607125..2609467 (-)	EFF01467 (Tde)	42.8	0	594	0.422
	LH408_RS12215	2612273..2612764 (-)	EFF00560 (-)	94.5	2.64E-109	303	0.945
	LH408_RS13360	2834745..2835197 (-)	EFF01516 (Tse)	50	1.90E-39	125	0.42
	LH408_RS13400	2840781..2845169 (-)	EFF01254 (Tse)	99.3	0	2988	0.993
	LH408_RS13425	2847818..2849740 (-)	EFF01840 (-)	84.6	0	1143	0.847
	LH408_RS13435	2850330..2852258 (-)	EFF01840 (-)	77.3	0	1040	0.773
	LH408_RS13465	2856452..2857870 (-)	EFF01872 (Tle)	99.6	0	967	0.996
	LH408_RS13525	2871855..2872337 (-)	EFF00505 (-)	100	2.10E-118	326	1
	LH408_RS13825	2939138..2939347 (-)	EFF01478 (Tse)	73.1	1.34E-33	104	0.71
	LH408_RS14315	3037839..3038330 (+)	EFF01309 (-)	66.2	6.61E-84	239	0.65
	LH408_RS17130	3638475..3638966 (+)	EFF01309 (-)	62	6.00E-74	214	0.62
E. cloacae POL9	LMJ45_RS00160	31751..32230 (+)	EFF00429 (-)	49.7	4.35E-61	181	0.503
	LMJ45_RS01025	213061..213273 (+)	EFF01477 (Tse)	76.1	1.07E-36	112	0.771
	LMJ45_RS02855	595938..596423 (+)	EFF01309 (-)	57.5	1.12E-68	200	0.571
	LMJ45_RS05780	1223688..1225073 (+)	EFF01485 (Tde)	52.8	1.22E-169	480	0.534
	LMJ45_RS06025	1278220..1282422 (+)	EFF01255 (Tse)	99	0	2586	0.904
	LMJ45_RS06365	1355908..1356117 (+)	EFF01478 (Tse)	72.1	3.48E-34	106	0.71
	LMJ45_RS07665	1622604..1624256 (-)	EFF01841 (-)	92.7	0	1073	0.927
	LMJ45_RS07705	1633709..1633930 (-)	EFF01478 (Tse)	54.7	1.42E-22	77	0.479
	LMJ45_RS10485	2209487..2209969 (-)	EFF01309 (-)	70	9.53E-87	246	0.7
	LMJ45_RS10635	2245551..2246033 (-)	EFF01309 (-)	69.4	8.22E-82	233	0.694
	LMJ45_RS11130	2355294..2356166 (-)	EFF01453 (Tse)	82.6	0	499	0.817
	LMJ45_RS11920	2517520..2517882 (+)	EFF00045 (Tae)	44.6	6.04E-30	99.4	0.45
	LMJ45_RS12325	2609966..2610457 (-)	EFF00560 (-)	94.5	2.64E-109	303	0.945

	LMJ45_RS13080	2771029..2772291 (-)	EFF01495 (Tre)	64.6	5.53E-80	248	0.448
	LMJ45_RS13090	2773100..2775028 (-)	EFF01840 (-)	77.4	0	1042	0.774
	LMJ45_RS13160	2789293..2789784 (-)	EFF01418 (Tae)	100	4.26E-124	340	1
	LMJ45_RS13165	2789843..2790325 (-)	EFF00505 (-)	100	2.10E-118	326	1
	LMJ45_RS13450	2855406..2855615 (-)	EFF01478 (Tse)	73.1	1.34E-33	104	0.71
E. cloacae MY490	HPB40_RS04165	854847..855326 (+)	EFF00429 (-)	50.3	1.18E-61	182	0.509
	HPB40_RS05035	1035878..1036090 (+)	EFF01477 (Tse)	76.1	1.07E-36	112	0.771
	HPB40_RS09795	2070744..2072129 (+)	EFF01485 (Tde)	52.6	6.63E-169	478	0.531
	HPB40_RS10060	2125792..2130048 (+)	EFF01255 (Tse)	100	0	2899	1
	HPB40_RS10390	2200436..2200645 (+)	EFF01478 (Tse)	72.1	3.48E-34	106	0.71
	HPB40_RS11670	2463152..2464804 (-)	EFF01841 (-)	92.5	0	1073	0.925
	HPB40_RS11705	2472967..2473188 (-)	EFF01478 (Tse)	54.7	1.42E-22	77	0.479
	HPB40_RS15375	3171152..3171634 (-)	EFF01309 (-)	69.4	4.12E-82	234	0.694
	HPB40_RS15785	3251036..3251908 (-)	EFF01453 (Tse)	82.2	0	499	0.814
	HPB40_RS17960	3707946..3708443 (-)	EFF01516 (Tse)	52.9	3.06E-50	154	0.448
	HPB40_RS18005	3714029..3718417 (-)	EFF01254 (Tse)	100	0	3011	1
	HPB40_RS18030	3721066..3722988 (-)	EFF01840 (-)	84.9	0	1146	0.85
	HPB40_RS18045	3724960..3726888 (-)	EFF01840 (-)	77.3	0	1039	0.773
	HPB40_RS18070	3730003..3731421 (-)	EFF01872 (Tle)	100	0	972	1
	HPB40_RS18125	3743866..3744357 (-)	EFF01418 (Tae)	100	4.26E-124	340	1
	HPB40_RS18130	3744416..3744898 (-)	EFF00505 (-)	100	2.10E-118	326	1
	HPB40_RS18415	3809980..3810189 (-)	EFF01478 (Tse)	73.1	1.34E-33	104	0.71
	HPB40_RS19165	3948406..3948888 (+)	EFF01309 (-)	69.4	4.05E-85	242	0.694
	HPB40_RS22095	4553545..4554036 (+)	EFF01309 (-)	61.4	6.38E-73	211	0.614
E. cloacae GX1Z-1L	J4G41_RS01645	334482..334730 (-)	EFF01874 (Tde)	53.6	1.83E-25	85.1	0.451
	J4G41_RS01930	398743..401295 (+)	EFF01467 (Tde)	77.5	0	1205	0.676
	J4G41_RS02965	603981..606320 (-)	EFF01467 (Tde)	75.9	0	1205	0.741
	J4G41_RS03600	748318..748530 (-)	EFF01477 (Tse)	76.1	1.07E-36	112	0.771
	J4G41_RS07525	1554654..1555139 (+)	EFF01309 (-)	70.6	4.49E-84	239	0.702
	J4G41_RS10310	2148598..2149983 (+)	EFF01485 (Tde)	52.8	2.40E-169	479	0.534
	J4G41_RS10600	2205495..2205986 (-)	EFF01309 (-)	69.9	2.53E-87	247	0.699
	J4G41_RS10630	2210812..2215068 (+)	EFF01255 (Tse)	98.7	0	2863	0.987
	J4G41_RS11035	2298197..2298406 (+)	EFF01478 (Tse)	72.1	3.48E-34	106	0.71
	J4G41_RS12565	2601562..2603214 (-)	EFF01841 (-)	92.7	0	1073	0.927
	J4G41_RS12600	2611377..2611598 (-)	EFF01478 (Tse)	54.7	1.42E-22	77	0.479
	J4G41_RS13245	2757701..2760022 (+)	EFF01467 (Tde)	83.7	0	1322	0.81
	J4G41_RS13280	2763984..2764196 (+)	EFF01895 (Tde)	48.6	1.69E-16	65.1	0.5
	J4G41_RS15990	3283017..3283499 (-)	EFF01309 (-)	69.4	1.85E-81	233	0.694

	J4G41_RS16295	3334128..3334643 (-)	EFF01309 (-)	57.7	1.77E-71	207	0.55
	J4G41_RS16520	3400141..3401013 (-)	EFF01453 (Tse)	82.2	0	495	0.814
	J4G41_RS18555	3817541..3818875 (-)	EFF01495 (Tre)	60.3	5.17E-148	424	0.608
	J4G41_RS18565	3819684..3821612 (-)	EFF01840 (-)	77.6	0	1042	0.776
	J4G41_RS18595	3825302..3826768 (-)	EFF01872 (Tle)	83.6	0	835	0.834
	J4G41_RS18650	3840052..3840534 (-)	EFF00505 (-)	100	2.10E-118	326	1
	J4G41_RS18740	3861113..3861592 (+)	EFF00429 (-)	54	6.14E-62	183	0.547
	J4G41_RS18955	3908105..3908314 (-)	EFF01478 (Tse)	73.1	1.34E-33	104	0.71
	J4G41_RS21920	4542373..4542855 (+)	EFF01309 (-)	61.6	1.21E-67	197	0.581
E. cloacae AVS0889	MBA36_RS00160	31672..32151 (+)	EFF00429 (-)	50.3	1.18E-61	182	0.509
	MBA36_RS00840	173762..173974 (+)	EFF01477 (Tse)	76.1	1.07E-36	112	0.771
	MBA36_RS05455	1169657..1171042 (+)	EFF01485 (Tde)	52.6	6.63E-169	478	0.531
	MBA36_RS05785	1234030..1238286 (+)	EFF01255 (Tse)	100	0	2899	1
	MBA36_RS06085	1305770..1305979 (+)	EFF01478 (Tse)	72.1	3.48E-34	106	0.71
	MBA36_RS07570	1599074..1600726 (-)	EFF01841 (-)	92.5	0	1073	0.925
	MBA36_RS07605	1608889..1609110 (-)	EFF01478 (Tse)	54.7	1.42E-22	77	0.479
	MBA36_RS11130	2313367..2314239 (-)	EFF01453 (Tse)	82.2	0	499	0.814
	MBA36_RS13350	2779702..2780199 (-)	EFF01516 (Tse)	52.9	3.06E-50	154	0.448
	MBA36_RS13395	2785785..2790173 (-)	EFF01254 (Tse)	100	0	3011	1
	MBA36_RS13420	2792822..2794744 (-)	EFF01840 (-)	84.9	0	1146	0.85
	MBA36_RS13435	2796716..2798644 (-)	EFF01840 (-)	77.3	0	1039	0.773
	MBA36_RS13460	2801759..2803177 (-)	EFF01872 (Tle)	100	0	972	1
	MBA36_RS13515	2815622..2816113 (-)	EFF01418 (Tae)	100	4.26E-124	340	1
	MBA36_RS13520	2816172..2816654 (-)	EFF00505 (-)	100	2.10E-118	326	1
	MBA36_RS13805	2881736..2881945 (-)	EFF01478 (Tse)	73.1	1.34E-33	104	0.71
	MBA36_RS14285	2980868..2981350 (+)	EFF01309 (-)	69.4	4.05E-85	242	0.694
	MBA36_RS17140	3589755..3590246 (+)	EFF01309 (-)	61.4	6.38E-73	211	0.614
	MBA36_RS17960	3768723..3769208 (-)	EFF01309 (-)	62.2	4.05E-75	216	0.634
E. cloacae 12961-yvys	-	-	-	-	-	-	-
E. cloacae SD21	MQH03_RS00160	32692..33171 (+)	EFF00429 (-)	50.3	1.18E-61	182	0.509
	MQH03_RS01020	214128..214340 (+)	EFF01477 (Tse)	76.1	1.07E-36	112	0.771
	MQH03_RS05280	1119041..1120426 (+)	EFF01485 (Tde)	52.8	2.43E-169	479	0.534
	MQH03_RS05520	1170775..1171266 (-)	EFF01309 (-)	69.3	2.44E-86	245	0.693
	MQH03_RS05550	1176119..1180318 (+)	EFF01255 (Tse)	99.1	0	2581	0.903
	MQH03_RS05885	1251679..1251888 (+)	EFF01478 (Tse)	72.1	3.48E-34	106	0.71
	MQH03_RS07195	1523681..1525333 (-)	EFF01841 (-)	92.5	0	1072	0.925
	MQH03_RS07230	1533496..1533717 (-)	EFF01478 (Tse)	54.7	1.42E-22	77	0.479
	MQH03_RS09140	1928730..1929221 (-)	EFF01309 (-)	60	1.76E-71	207	0.589

	MQH03_RS10260	2153857..2154348 (-)	EFF01309 (-)	58.3	8.96E-73	211	0.583
	MQH03_RS10475	2219943..2220815 (-)	EFF01453 (Tse)	82.6	0	499	0.817
	MQH03_RS11070	2344490..2344972 (+)	EFF01309 (-)	69.4	2.47E-85	242	0.694
	MQH03_RS11275	2387628..2387990 (+)	EFF00045 (Tae)	44.6	6.04E-30	99.4	0.45
	MQH03_RS11655	2469128..2471911 (-)	EFF01467 (Tde)	84.4	0	1325	0.683
	MQH03_RS11740	2491515..2493872 (-)	EFF01467 (Tde)	53.3	0	788	0.498
	MQH03_RS11750	2496694..2497185 (-)	EFF00560 (-)	94.5	5.63E-110	305	0.945
	MQH03_RS12540	2661377..2665810 (-)	EFF01254 (Tse)	98	0	2674	0.884
	MQH03_RS12565	2668459..2670381 (-)	EFF01840 (-)	84.6	0	1144	0.847
	MQH03_RS12575	2670971..2672899 (-)	EFF01840 (-)	77.4	0	1042	0.774
	MQH03_RS12645	2687164..2687655 (-)	EFF01418 (Tae)	100	4.26E-124	340	1
	MQH03_RS12650	2687714..2688196 (-)	EFF00505 (-)	100	2.10E-118	326	1
	MQH03_RS12940	2754192..2754401 (-)	EFF01478 (Tse)	73.1	1.34E-33	104	0.71
	MQH03_RS15935	3396486..3396977 (+)	EFF01309 (-)	61.4	8.06E-74	213	0.614
	MQH03_RS19300	4098862..4101414 (+)	EFF01467 (Tde)	78	0	1214	0.681
E. cloacae EFN743	MKZ88_RS01465	288234..288446 (+)	EFF01477 (Tse)	76.1	1.07E-36	112	0.771
	MKZ88_RS10250	2085169..2085651 (-)	EFF01309 (-)	69.4	4.05E-85	242	0.694
	MKZ88_RS10725	2182346..2182555 (+)	EFF01478 (Tse)	73.1	1.34E-33	104	0.71
	MKZ88_RS10940	2229103..2229582 (-)	EFF00429 (-)	54	2.29E-62	184	0.547
	MKZ88_RS11020	2249330..2249812 (+)	EFF00505 (-)	100	2.10E-118	326	1
	MKZ88_RS11025	2249871..2250362 (+)	EFF01418 (Tae)	100	4.26E-124	340	1
	MKZ88_RS11080	2262807..2264273 (+)	EFF01872 (Tle)	83.6	0	835	0.834
	MKZ88_RS11110	2267186..2269114 (+)	EFF01840 (-)	77.6	0	1043	0.776
	MKZ88_RS11120	2269704..2271626 (+)	EFF01840 (-)	84.9	0	1147	0.85
	MKZ88_RS11145	2274275..2278651 (+)	EFF01254 (Tse)	98.3	0	2692	0.896
	MKZ88_RS11940	2446720..2447211 (+)	EFF00560 (-)	94.5	2.64E-109	303	0.945
	MKZ88_RS12505	2575842..2576324 (-)	EFF01309 (-)	69.4	3.43E-85	242	0.694
	MKZ88_RS13140	2705366..2706238 (+)	EFF01453 (Tse)	82.6	0	499	0.817
	MKZ88_RS13670	2828868..2829350 (+)	EFF01309 (-)	69.4	4.12E-82	234	0.694
	MKZ88_RS13830	2865870..2866352 (+)	EFF01309 (-)	70	9.53E-87	246	0.7
	MKZ88_RS14625	3036408..3038930 (-)	EFF01826 (Tse)	63.1	0	1061	0.624
	MKZ88_RS14645	3042828..3043319 (-)	EFF00560 (-)	93.9	3.80E-109	303	0.939
	MKZ88_RS14810	3073848..3074339 (+)	EFF01309 (-)	59.4	4.37E-71	206	0.583
	MKZ88_RS17275	3564785..3565006 (+)	EFF01478 (Tse)	54.7	1.42E-22	77	0.479
	MKZ88_RS17310	3573169..3574821 (+)	EFF01841 (-)	92.7	0	1073	0.927
	MKZ88_RS18800	3873640..3873849 (-)	EFF01478 (Tse)	72.1	3.48E-34	106	0.71
	MKZ88_RS19505	4024234..4025619 (-)	EFF01485 (Tde)	52.8	2.43E-169	479	0.534
	MKZ88_RS22755	4726726..4727211 (-)	EFF01309 (-)	70	4.69E-83	236	0.696

E. cloacae 3143	M5S62_RS00160	32692..33171 (+)	EFF00429 (-)	49.7	2.44E-61	181	0.503
	M5S62_RS00490	98695..99174 (-)	EFF01309 (-)	48.1	4.78E-56	168	0.484
	M5S62_RS01035	215830..216042 (+)	EFF01477 (Tse)	76.1	1.07E-36	112	0.771
	M5S62_RS05455	1162675..1164060 (+)	EFF01485 (Tde)	52.8	3.97E-169	479	0.534
	M5S62_RS05695	1217203..1221450 (+)	EFF01255 (Tse)	99.1	0	2587	0.896
	M5S62_RS06005	1292641..1292850 (+)	EFF01478 (Tse)	72.1	3.48E-34	106	0.71
	M5S62_RS07280	1554726..1556378 (-)	EFF01841 (-)	92.5	0	1071	0.925
	M5S62_RS07315	1564541..1564762 (-)	EFF01478 (Tse)	54.7	1.42E-22	77	0.479
	M5S62_RS10790	2240364..2240846 (-)	EFF01309 (-)	70	9.53E-87	246	0.7
	M5S62_RS11420	2392481..2393353 (-)	EFF01453 (Tse)	82.6	0	499	0.817
	M5S62_RS12005	2515185..2515667 (+)	EFF01309 (-)	70	1.86E-85	243	0.7
	M5S62_RS12585	2647247..2647738 (-)	EFF00560 (-)	94.5	2.64E-109	303	0.945
	M5S62_RS12780	2691108..2691320 (-)	EFF01478 (Tse)	52.2	2.15E-19	68.9	0.5
	M5S62_RS12785	2691604..2691822 (+)	EFF01478 (Tse)	60.6	1.23E-28	92.4	0.597
	M5S62_RS13790	2880248..2880739 (-)	EFF01516 (Tse)	50.6	4.40E-51	155	0.503
	M5S62_RS13800	2882237..2882422 (-)	EFF01254 (Tse)	100	3.02E-38	127	1
	M5S62_RS13890	2896070..2897992 (-)	EFF01840 (-)	84.7	0	1144	0.848
	M5S62_RS13900	2898582..2900510 (-)	EFF01840 (-)	77.3	0	1040	0.773
	M5S62_RS13930	2904704..2906122 (-)	EFF01872 (Tle)	99.6	0	967	0.996
	M5S62_RS13985	2918567..2919058 (-)	EFF01418 (Tae)	100	4.26E-124	340	1
	M5S62_RS13990	2919117..2919599 (-)	EFF00505 (-)	100	2.10E-118	326	1
	M5S62_RS14275	2984673..2984882 (-)	EFF01478 (Tse)	73.1	1.34E-33	104	0.71
	M5S62_RS15580	3263178..3264143 (+)	EFF01504 (Tse)	43.6	1.47E-85	255	0.436
E. cloacae 2022CK-00409	N7S94_RS00950	185149..185640 (-)	EFF00560 (-)	94.5	2.64E-109	303	0.945
	N7S94_RS01640	320787..321659 (+)	EFF01453 (Tse)	82.5	0	497	0.814
	N7S94_RS03690	749751..754184 (-)	EFF01254 (Tse)	98.4	0	2694	0.887
	N7S94_RS03725	757899..759821 (-)	EFF01840 (-)	85	0	1149	0.852
	N7S94_RS03735	760410..762338 (-)	EFF01840 (-)	77.8	0	1037	0.779
	N7S94_RS03775	767395..768861 (-)	EFF01872 (Tle)	83.4	0	832	0.832
	N7S94_RS03830	781306..781797 (-)	EFF01418 (Tae)	100	4.26E-124	340	1
	N7S94_RS03835	781856..782338 (-)	EFF00505 (-)	100	2.10E-118	326	1
	N7S94_RS04125	849346..849555 (-)	EFF01478 (Tse)	73.1	1.34E-33	104	0.71
	N7S94_RS13170	2674041..2674253 (-)	EFF01477 (Tse)	76.1	1.07E-36	112	0.771
	N7S94_RS13790	2804777..2804899 (+)	EFF01505 (Tse)	75	7.68E-10	45.8	0.525
	N7S94_RS16330	3347117..3349684 (+)	EFF01826 (Tse)	46.9	0	680	0.444
	N7S94_RS20480	4198202..4199587 (+)	EFF01485 (Tde)	52.8	2.43E-169	479	0.534
	N7S94_RS20730	4253973..4257776 (+)	EFF01255 (Tse)	99.4	0	2572	0.994
	N7S94_RS21085	4331766..4331975 (+)	EFF01478 (Tse)	72.1	3.48E-34	106	0.71

	N7S94_RS22375	4594405..4596057 (-)	EFF01841 (-)	92.7	0	1073	0.927
	N7S94_RS22415	4605420..4605641 (-)	EFF01478 (Tse)	54.7	1.42E-22	77	0.479

[a] Predicted effectors include putative cargo effectors and components of the puncturing device (Hcp, VgrG, and PAAR), which may possess toxin domains.

[b] The EFF designation denotes the SecReT6 database effector IDs with the best match to the query.

[c] The known or predicted function of each effector hit by SecReT6 is noted in parenthesis; (-), indicates an effector within the SecReT6 database with no assigned function.
